# Ferulic Acid Administered at Various Time Points Protects against Cerebral Infarction by Activating p38 MAPK/p90RSK/CREB/Bcl-2 Anti-Apoptotic Signaling in the Subacute Phase of Cerebral Ischemia-Reperfusion Injury in Rats

**DOI:** 10.1371/journal.pone.0155748

**Published:** 2016-05-17

**Authors:** Chin-Yi Cheng, Nou-Ying Tang, Shung-Te Kao, Ching-Liang Hsieh

**Affiliations:** 1 School of Chinese Medicine, College of Chinese Medicine, China Medical University, Taichung, 40402, Taiwan; 2 Department of Chinese Medicine, Hui-Sheng Hospital, 42056, Taichung, Taiwan; 3 Department of Chinese Medicine, China Medical University Hospital, 40447, Taichung, Taiwan; 4 Graduate Institute of Integrated Medicine, College of Chinese Medicine, China Medical University, Taichung, 40402, Taiwan; Indian Institute of Integrative Medicine, INDIA

## Abstract

**Objectives:**

This study aimed to evaluate the effects of ferulic acid (FA) administered at various time points before or after 30 min of middle cerebral artery occlusion (MCAo) followed by 7 d of reperfusion and to examine the involvement of mitogen-activated protein kinase (MAPK) signaling pathways in the cortical penumbra.

**Methods:**

FA was intravenously administered to rats at a dose of 100 mg/kg 24 h before ischemia (B-FA), 2 h before ischemia (P-FA), immediately after ischemic insult (I-FA), 2 h after reperfusion (R-FA), or 24 h after reperfusion (D-FA).

**Results:**

Our study results indicated that P-FA, I-FA, and R-FA effectively reduced cerebral infarct areas and neurological deficits. P-FA, I-FA, and R-FA significantly downregulated glial fibrillary acidic protein (GFAP), mitochondrial Bax, cytochrome c, and cleaved caspase-3 expression, and effectively restored the phospho-p38 MAPK (p-p38 MAPK)/p38 MAPK ratio, phospho-90 kDa ribosomal S6 kinase (p-p90RSK) expression, phospho-Bad (p-Bad) expression, the phospho-cAMP response element-binding protein (p-CREB)/CREB ratio, the cytosolic and mitochondrial Bcl-2/Bax ratios, and the cytosolic Bcl-xL/Bax ratio in the cortical penumbra 7 d after reperfusion. SB203580, a specific inhibitor of p38 MAPK, administered 30 min prior to ischemia abrogated the downregulating effects of I-FA on cerebral infarction, and mitochondrial Bax and cleaved caspase-3 expression, and the upregulating effects of I-FA on the p-p38 MAPK/p38 MAPK ratio, p-p90RSK expression, p-Bad expression, and the p-CREB/CREB, and cytosolic and mitochondrial Bcl-2/Bax ratios.

**Conclusions:**

Our study results thus indicate that P-FA, I-FA, and R-FA effectively suppress reactive astrocytosis and exert neuroprotective effects against cerebral infarction by activating p38 MAPK signaling. The regulating effects of P-FA, I-FA, and R-FA on Bax-induced apoptosis result from activation of the p38 MAPK/p90RSK/CREB/Bcl-2 signaling pathway, and eventually contribute to inhibition of the cytochrome c-mediated caspase-3-dependent apoptotic pathway in the cortical penumbra 7 d after reperfusion.

## Introduction

Mitogen-activated protein kinases (MAPKs) comprise three major members, c-Jun N-terminal kinase (JNK), extracellular signal-regulated kinase 1/2 (ERK1/2), and p38 MAPK, which convey extracellular signals to their intracellular targets to regulate cellular activities through various signaling pathways [1,2]. The p38 MAPK pathway might play distinct roles in various phases of cerebral ischemia. Studies have demonstrated that sustained activation of p38 MAPK exacerbates cerebral infarction by promoting inflammatory responses in the acute phase after transient middle cerebral artery occlusion (MCAo) [3,4]. Studies have also shown that phosphorylated p38 MAPK exerts neuroprotective effects against apoptosis in the penumbral cortex during the acute [5] and subacute phases [6] of cerebral ischemia. The pharmacological inhibition of p38 MAPK increases brain injury and vascular leakage in a rat model of transient MCAo [2]. ERK1/2 activation directly phosphorylates 90 kDa ribosomal S6 kinase (p90RSK), which subsequently phosphorylates the pro-apoptotic protein Bad, resulting in protection against apoptosis in rat models of transient focal cerebral ischemia [7,8]. Previous studies have suggested that p90RSK might also play a crucial role in the crosstalk between ERK1/2 and p38 MAPK signaling pathways in in vitro [9] and in vivo [10] models. Lian et al. (1999) showed that the p38 MAPK inhibitor SB203580, at higher concentrations, inhibits the activation of ERK1/2 and p90RSK in stimulated neutrophils, indicating a close relationship between p38 MAPK and p90RSK signaling cascades [11]. The ERK1/2 and p38 MAPK signaling pathways activate the transcription factor cyclic AMP response element (CRE) binding protein (CREB) (Ser 133) to promote neuronal survival in the ischemic area during the reperfusion period after focal cerebral ischemia [6,12]. CREB regulates several downstream genes containing CRE sequences and plays crucial roles in cell proliferation, differentiation, adaption, and survival [13,14]. CREB phosphorylation upregulates CRE-mediated genes including Bcl-2 and Bcl-xL, which provide neuroprotective effects against apoptosis by preserving the integrity of the outer mitochondrial membrane in the ischemic cortex after transient MCAo [6,15]. The Bcl-2 family members include antiapoptotic (Bcl-2 and Bcl-xL) and proapoptotic (Bax) proteins, and the ratio of Bcl-2(Bcl-xL)/Bax determines whether ischemic neurons undergo death or survival after an apoptotic stimulus [16]. Accumulating evidence has indicated that an increased ratio of Bcl-2(Bcl-xL)/Bax prevents the activation and translocation of Bax to the mitochondria, leading to antiapoptosis, whereas a decreased Bcl-2(Bcl-xL)/Bax ratio induces mitochondrial Bax homo-oligomerization, leading to the formation of pores in the mitochondrial outer membrane, and subsequent activation of the cytochrome c-mediated apoptotic cascade in the ischemic area after cerebral ischemia [16–18].

Ferulic acid (4-hydroxy-3-methoxycinnamic acid, FA) is the major active component in *Angelica sinensis* (Oliv.) Diels (AS) and *Ligusticum chuanxiong* Hort (LC). AS and LC have both been used to treat stroke in traditional Chinese medicine [19,20]. Our previous studies showed that FA is neuroprotective against cerebral infarction by inhibiting inflammation and oxidative stress in the ischemic area 24 h after transient MCAo [21,22]. Other studies have shown that FA protects against caspase-3-mediated apoptosis by regulating MAPK or phosphatidylinositol-3-kniase (PI3K)/Akt signaling pathways in the acute phase of focal cerebral ischemia [23,24], and in an aged rat model [25]. Zhang et al. (2015) indicated that FA treatment ameliorates ischemic hippocampal neuronal injury by increasing erythropoietin expression in the ischemic region 7 d after transient MCAo [20]. However, the therapeutic time window and precise mechanisms underlying the neuroprotective effect of FA against ischemic injury in the subacute phase of mild transient MCAo remain unclear.

Therefore, this study aimed to evaluate the effects of FA administered at various time points before or after 30 min of cerebral ischemia followed by 7 d of reperfusion, and to evaluate the involvement of MAPK signaling pathways in the ischemic cortical penumbra.

## Materials and Methods

### Experimental Animals

Male Sprague Dawley (SD) rats weighing 300–350 g (aged approximately 8–9 wk) were purchased from Lasco Co. (Taiwan). They were maintained at a humidity of 55% ± 5% on a 12 h light-dark cycle at 22 ± 2°C.

### Ethics Statement

All experimental procedures were performed in strict accordance with the ethical guidelines provided by the China Medical University Institutional Animal Care and Use Committee (Permit Number: 103-33-N), and the committee recognized that the methodologies and study designs followed the Animal Protection Law by the Council of Agriculture, Executive Yuan, Taiwan. All procedures involving animals avoided or minimized discomfort, pain, and stress in the animals.

### Transient Middle Cerebral Artery Occlusion

The MCAo model was established in the SD rats by using the intraluminal suture technique as described previously [26]. Briefly, the rats were anesthetized with a 5% isoflurane-oxygen mixture, and then maintained on a 2% isoflurane-oxygen mixture. The head of the rat was fixed in a stereotactic frame and the right distal middle cerebral artery (MCA) was exposed through a cranial burr hole (2.5 mm lateral and 2.0 mm posterior to the bregma). After neck dissection, the right common carotid artery (CCA) and internal carotid artery (ICA) were exposed, and the pterygopalatine artery was ligated close to its origin. A 3–0 nylon suture with a blunt tip was inserted into the ICA until the tip occluded the origin of the MCA. After 30 min of MCAo, the suture was carefully withdrawn to permit reperfusion. Blood flow in the MCA was monitored using Laser-Doppler flowmetry (DRT4, Moor Instruments Inc, Wilmington, USA) through the cranial burr hole in the preischemia (>500 units), ischemia (<100 units), and reperfusion (>300 units) periods. These results were used to confirm the success of the animal model.

### Evaluation of Neurological Status

The neurological function of each rat was evaluated 1, 3, and 7 d after reperfusion. Motor, sensory, balance, and reflex functions were determined using modified neurological severity scores, as described previously [27]. The overall neurological function of each rat was graded according to a numerical scale from 0 to 18 (reference score, 0; maximal deficit score, 18).

### Experiment A

#### Grouping

The rats were randomly divided into Sham, Vehicle, B-FA, P-FA, I-FA, R-FA, and D-FA groups (n = 6). The rats in the I-FA group were subjected to MCAo and immediately intravenously administered FA (Sigma-Aldrich) at a dose of 100 mg/kg. After 30 min of ischemia, the rats were subjected to reperfusion and sacrificed 7 d after reperfusion. The rats in the B-FA group were subjected to the same procedures as the rats in the I-FA group; however, FA was administered 1 d before cerebral ischemia. The rats in the P-FA group were subjected to the same procedures as the rats in the I-FA group; however, FA was administered 2 h before cerebral ischemia. The rats in the R-FA group were subjected to the same procedures as the rats in the I-FA group; however FA was administered 2 h after reperfusion. The rats in the D-FA group were subjected to the same procedures as the rats in the I-FA group; however, FA was administered 1 d after reperfusion. The rats in the Vehicle group were subjected to the same procedures as the rats in the I-FA group, but did not receive FA. The rats in the Sham group were subjected to the same procedures as the rats in the Vehicle group, but the MCA origin was not occluded.

#### Evaluation of cerebral infarction

Following neurological evaluation 1, 3, and 7 d after reperfusion, the rats were sacrificed. Their brains were removed immediately and cut into 6 coronal sections of 2-mm thickness. The coronal brain sections were immersed with 2% 2,3,5-triphenyltetrazolium chloride (TTC; Merck, Germany) for 5 min at 37°C. The brain tissues were differentiated according to white-colored infarct and dark red noninfarct areas, and the cerebral infarct areas were determined using image analysis software (ImageJ, Java). The ratios of cerebral infarction area to total brain area were then calculated.

### Experiment B

#### Grouping

The rats were randomly divided into 7 groups: Sham, Vehicle, B-FA, P-FA, I-FA, R-FA, and D-FA groups (n = 4–5). They were then subjected to the experimental procedures described in Experiment A.

#### Western Blot Analysis

After 7 d of reperfusion, the rats were sacrificed, and the brains were immediately removed and coronally sectioned from -4.3 to +1.7 mm bregma. The right ischemic frontoparietal cortex between 3 and 8 mm from the ischemic core was considered as the penumbra fraction. The right penumbral cortex was further divided into cytosolic and mitochondrial fractions according to the manufacturer’s instructions (#K256-100 BioVision, USA), and protein levels in the cytosolic and mitochondrial fractions were determined using a Bio-Rad protein assay. The protein samples were subjected to sodium dodecyl sulfate-polyacrylamide gel electrophoresis and then transferred to a nitrocellulose membrane in Western blot analysis as described previously [28]. After transfer, the membrane was incubated with a rabbit anti-p38 MAP kinase (p38 MAPK; 1/1000 dilution, #9212 Cell Signaling), rabbit anti-phospho-p38 MAPK (p-p38 MAPK (Thr180/Tyr182); 1/1000 dilution, #9211 Cell Signaling), rabbit anti-p44/42 MAPK (ERK1/2; 1/1000 dilution, #9102 Cell Signaling), rabbit anti-phospho-p44/42 MAPK (p-ERK1/2; 1/1000 dilution, #9101 Cell Signaling), rabbit anti-SAPK/JNK (JNK; 1/1000 dilution, #9252 Cell Signaling), rabbit anti-phospho-SAPK/JNK (p-JNK (Thr183/Tyr185); 1/1000 dilution, #9251 Cell Signaling), rabbit anti-Akt (1/1000 dilution, #4685 Cell Signaling), rabbit anti-phospho-Akt (p-Akt (Ser473); 1/1000 dilution, #9271 Cell Signaling), mouse anti-glial fibrillary acidic protein (GFAP; 1/1000 dilution, #3670 Cell Signaling), rabbit anti-heat shock protein 70 (HSP70; 1/1000 dilution, #4872 Cell Signaling), rabbit anti-CREB (1/1000 dilution, #9197 Cell Signaling), mouse anti-phospho-CREB (p-CREB; 1/500 dilution, DAM1482729 Millipore), rabbit anti-phospho-p90RSK (p-p90RSK; 1/1000 dilution, #9344 Cell Signaling), rabbit anti-phospho-Bad (p-Bad; 1/1000 dilution, #9291 Cell Signaling), rabbit anti-Bcl-2 (1/1000 dilution, #2876 Cell Signaling), rabbit anti-Bax (1/1000 dilution, #2772 Signaling), rabbit anti-Bcl-xL (1/1000 dilution, #2762 Cell Signaling), rabbit anti-apoptosis-inducing factor (AIF; 1/1000 dilution, #4642 Cell Signaling), or rabbit anti-cleaved caspase-3 (1/1000 dilution, #9661S Cell Signaling) antibody overnight at 4°C. The membranes containing the transferred proteins were also probed with antibodies specific for mouse anti-actin (1/5000 dilution, MAB1501 Chemicon) and rabbit anti-heat shock protein 60 (HSP60; 1/1000 dilution, #4870 Cell Signaling), as internal controls for the cytosolic and mitochondrial fractions, respectively. After washing the membranes 4 times in Tris-buffered saline (TBS), the membranes were incubated with an anti-rabbit horseradish peroxidase (HRP)-conjugated IgG (1/5000 dilution, Jackson ImmunoResearch) or anti-mouse HRP-conjugated IgG (1/5000 dilution, Santa Cruz Biotechnology) antibody for 1 h at room temperature (RT). The blots were then incubated with an enhanced chemiluminescence plus reagent solution (ECL-plus GE Healthcare) on a luminescence image analyzer (LAS-3000, FujiFilm). Densitometric analysis of the images was performed using ImageJ software. Results were expressed as optical density ratios of proteins to actin or HSP60.

#### Immunohistochemical analysis

After 30 min of cerebral ischemia followed by 7 d of reperfusion, the rats were sacrificed (n = 3). They were transcardially perfused with saline, and their brains were removed, postfixed in paraformalaldehyde, cut into sections, and incubated with normal animal serum as described previously [29]. The brain sections were then incubated in a moist chamber with a rabbit anti-cytochrome c (1/50 dilution, #10993-1-AP proteintech) or rabbit anti-cleaved caspase-3 (1/200 dilution, #9664 Cell Signaling) antibody overnight at 4°C. Following incubation with the appropriate secondary antibody and avidin-biotin peroxidase complexes (ABC kit, ScyTek, Logan, Utah, USA), the sections were colored using a 3,3’-diaminobenzidine (DAB) kit (ScyTek, Logan, Utah, USA), and counterstained with hematoxylin. The stained sections were then mounted in a mounting medium (Assistant-Histokitt, Germany), and the cytochrome c- and cleaved caspase-3-positive cells in the ischemic penumbral cortex were analyzed using a light microscope (Axioskop 40, Zeiss). Samples from the Vehicle group stained without the cytochrome c or cleaved caspase-3 primary antibody were used as negative controls.

#### Immunofluorescent costaining

The brain sections adjacent to those used in immunohistochemical (IHC) analysis were blocked in 10% normal animal serum (ScyTek, Logan, Utah, USA) for 20 min at RT. They were subsequently incubated with a mouse anti-p-CREB (1/100 dilution, DAM1482729 Millipore) antibody overnight at 4°C. After washing 3 times with phosphate buffered saline (PBS) containing 0.1% (v/v) Tween-20 (TPBS), the brain sections were incubated with a DyLight 594-conjugated AffiniPure goat anti-mouse IgG antibody (red, 1/400 dilution, Jackson ImmunoResearch) for 1 h at RT. The p-CREB-stained sections were then counterstained with 4',6-diamidino-2-phenylindole (DAPI; 1/100 dilution, Sigma-Aldrich, USA, nuclear staining) for 20 min at RT. All sections were mounted in an aqueous mounting medium (Aquatex, HC886685 Merck) and the cortical periinfarct regions were examined with a fluorescent microscope (CKX41, Olympus). Sections from the I-FA group incubated without the p-CREB primary antibody were analyzed as negative controls.

### Experiment C

#### Grouping

The rats were randomly divided into D+Sham, D+Vehicle, D+I-FA, and SB+I-FA groups (n = 3). The rats in the SB+I-FA group were subjected to the same procedures as the rats in the I-FA group (experiment A), but also received an intracerebroventricular (ICV) injection of the p38 MAPK inhibitor SB203580 30 min prior to the onset of MCAo. The rats in the D+I-FA group were subjected to the same procedures as the rats in the SB+I-FA group, but received an ICV injection of 1% dimethyl sulfoxide (DMSO). The rats in the D+Vehicle group were subjected to the same procedures as the rats in the Vehicle group (experiment A), but also received an ICV injection of 1% DMSO 30 min prior to the onset of MCAo. The rats in the D+Sham group were subjected to the same procedures as the rats in the D+Vehicle group, but the MCA origin was not occluded.

#### Intracerebroventricular injection of SB203580 or 1% dimethyl sulfoxide

The rats were anesthetized with a 2% isoflurane-oxygen mixture and their heads were fixed in a stereotactic frame. Five microliters (μl) of a solution containing SB203580 (1 mM in 1% DMSO, #S1076 Selleckchem.com) or 1% DMSO were administered by ICV injection to the right hemisphere. The injections were performed using a 5-μl Hamilton syringe with a 26-gauge needle (Hamilton Company, Nevada, USA). The location of each injection was 0.8 mm posterior to the bregma, 1.5 mm lateral to the midline, and 3.5 mm deep into the skull surface.

#### Evaluation of cerebral infarction

Seven days after reperfusion, the rats were sacrificed. They were then subjected to the experimental procedures of cerebral infarct evaluation described in Experiment A.

### Experiment D

#### Grouping

The rats were randomly divided into 4 groups: D+Sham, D+Vehicle, D+I-FA, and SB+I-FA groups (n = 5). They were then subjected to the experimental procedures described in Experiment C.

#### Western Blot Analysis

After 7 d of reperfusion, the rats were sacrificed, and their brains were removed for Western blot analysis of p38 MAPK, p-p38 MAPK, p-p90RSK, p-Bad, CREB, p-CREB, Bcl-2, Bax, and cleaved caspase-3 expression. The samples were subjected to Western blotting procedures as described in Experiment B.

### Statistical Analysis

Data are expressed as mean ± standard deviation (SD). Data from all experimental groups were compared using a one-way analysis of variance (ANOVA) followed by post-hoc analysis by using the Scheffe test. *P* < 0.05 was considered statistically significant.

## Results

### Effects of P-FA, I-FA, and R-FA on Cerebral Infarct Area

**Fig 1:**
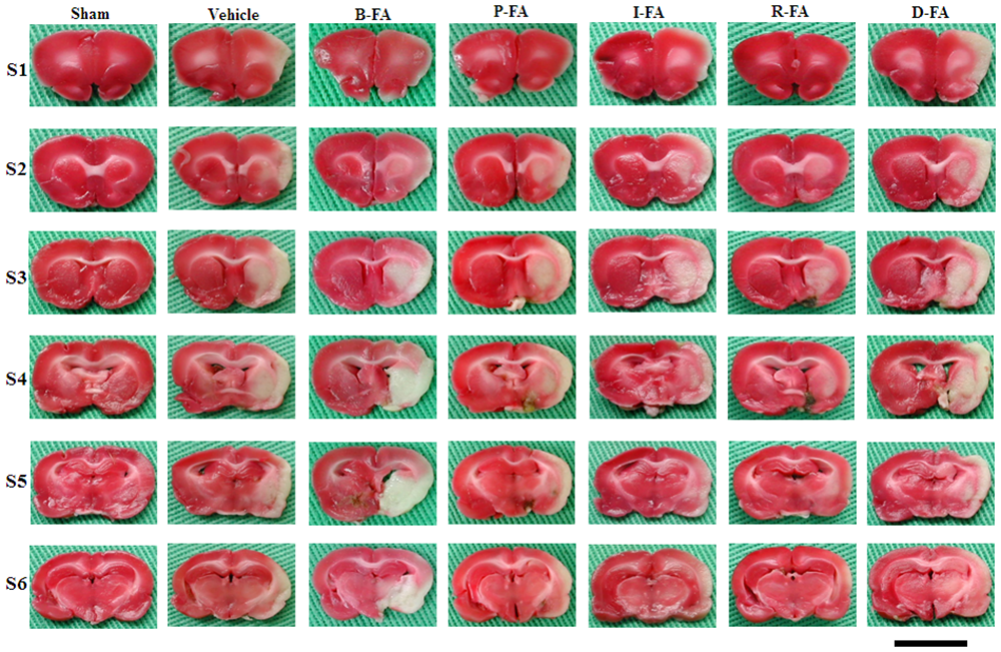
Cerebral infarction (S1–S6) among the experimental groups after 30 min of MCAo followed by 7 d of reperfusion. 2,3,5-Triphenyltetrazolium chloride (TTC) staining shows normal brain tissue (deep red) and infarct tissue (white). Scale bar = 1 cm.

**Fig 2:**
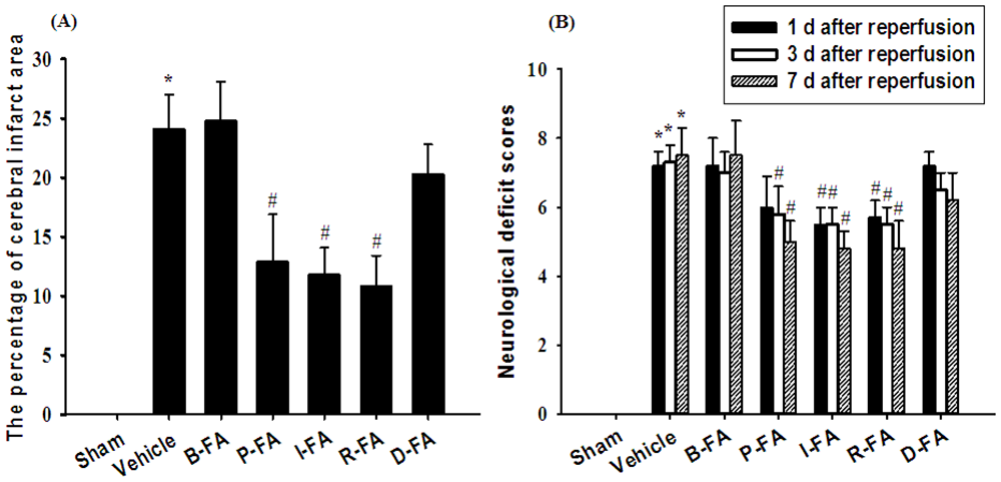
Effects of P-FA, I-FA, and R-FA on cerebral ischemic areas and neurological behaviors 7 d after reperfusion. (A) The percentage cerebral infarct areas in the Sham, Vehicle, B-FA, P-FA, I-FA, R-FA, and D-FA groups were evaluated 7 d after reperfusion (n = 6). (B) The neurological deficit scores among the Sham, Vehicle, B-FA, P-FA, I-FA, R-FA, and D-FA groups were examined 1, 3, and 7 d after reperfusion. Data are presented as mean ± SD. *P < 0.05 versus the Sham group; #*P* < 0.05 versus the Vehicle group.

Seven days after reperfusion, the rats developed prominent cerebral infarction throughout the MCA territory involving the cortex and striatum (*P* < 0.05 vs. Sham group; Figs 1 and 2A). The percentage cerebral infarct areas were significantly lower in the P-FA, I-FA, and R-FA groups than in the Vehicle group (all *P* < 0.05; Figs 1 and 2A). However, the percentage cerebral infarct areas in the Vehicle, B-FA, and D-FA groups showed nonsignificant differences (*P* > 0.05).

### Effects of P-FA, I-FA, and R-FA on Neurological Status

One day after reperfusion, the rats were evaluated for the extent of neurological impairment and graded as having moderate neurological deficit scores (approximately 5.5–7.2). The neurological deficit scores of the I-FA and R-FA groups were significantly lower than those of the Vehicle group (both *P* < 0.05; Fig 2B). The neurological deficit scores of the Vehicle, B-FA, P-FA, and D-FA groups showed nonsignificant differences (*P* > 0.05). Three days after reperfusion, the neurological deficit scores of the P-FA, I-FA and R-FA groups were significantly lower than those of the Vehicle group (all *P* < 0.05; Fig 2B), whereas the neurological deficit scores of the Vehicle, B-FA, and D-FA groups showed nonsignificant differences (*P* > 0.05). Seven days after reperfusion, the neurological deficit scores of the P-FA, I-FA, and R-FA groups were significantly lower than those of the Vehicle groups (all *P* < 0.05; Fig 2B). However, the neurological deficit scores of the Vehicle, B-FA, and D-FA groups showed nonsignificant differences (*P* > 0.05).

### Effects of P-FA, I-FA, and R-FA on the Cytosolic Expression of p-MAPKs, MAPKs, p-Akt, and Akt

**Fig 3:**
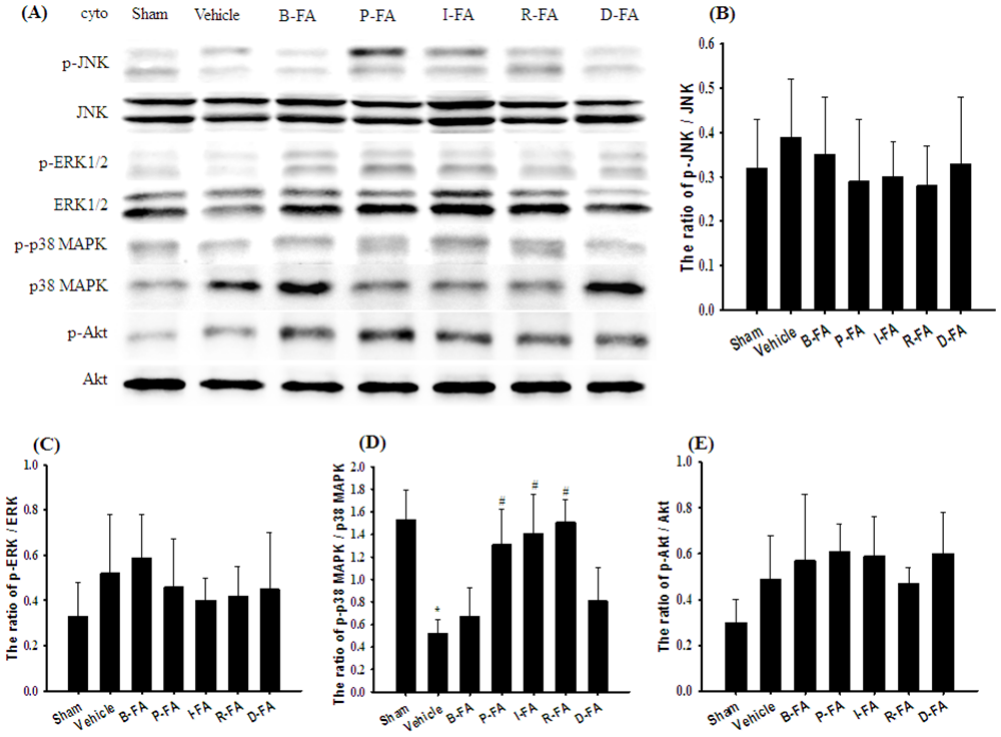
Effects of P-FA, I-FA, and R-FA on the cytosolic expression of p-JNK, JNK, p-ERK1/2, ERK1/2, p-p38 MAPK, p38 MAPK, p-Akt, and Akt in the cortical penumbra. (A) Representative Western blot images show cytosolic p-JNK, JNK, p-ERK1/2, ERK1/2, p-p38 MAPK, p38 MAPK, p-Akt, and Akt expression in the cortical penumbra in the Sham, Vehicle, B-FA, P-FA, I-FA, R-FA, and D-FA groups 7 d after reperfusion. The ratios of (B) p-JNK/JNK, (C) p-ERK/ERK, (D) p-p38 MAPK/p38 MAPK, and (E) p-Akt/Akt expression were calculated in the cortical penumbra in the Sham, Vehicle, B-FA, P-FA, I-FA, R-FA, and D-FA groups (n = 5). cyto, cytosolic fraction. Data are presented as mean ± SD. *P < 0.05 versus the Sham group; #*P* < 0.05 versus the Vehicle group.

Western blot analysis indicated that the ratios of cytosolic p-JNK/JNK, p-ERK/ERK, and p-Akt/Akt in the cortical penumbra among the experimental groups showed nonsignificant differences 7 d after reperfusion (*P* > 0.05; Fig 3A, 3B, 3C and 3E). The ratio of cytosolic p-p38 MAPK/p38 MAPK expression was significantly lower in the Vehicle group (0.3-fold) than in the Sham group (*P* < 0.05), and significantly higher in the P-FA (2.5-fold), I-FA (2.7-fold), and R-FA (2.9-fold) groups than in the Vehicle group (all *P* < 0.05; Fig 3A and 3D). The ratio of cytosolic p-p38 MAPK/p38 MAPK expression in the Vehicle, B-FA, and D-FA groups showed nonsignificant differences (*P* > 0.05).

### Effects of P-FA, I-FA, and R-FA on the Cytosolic Expression of HSP70, GFAP, p-p90RSK, and p-Bad

**Fig 4:**
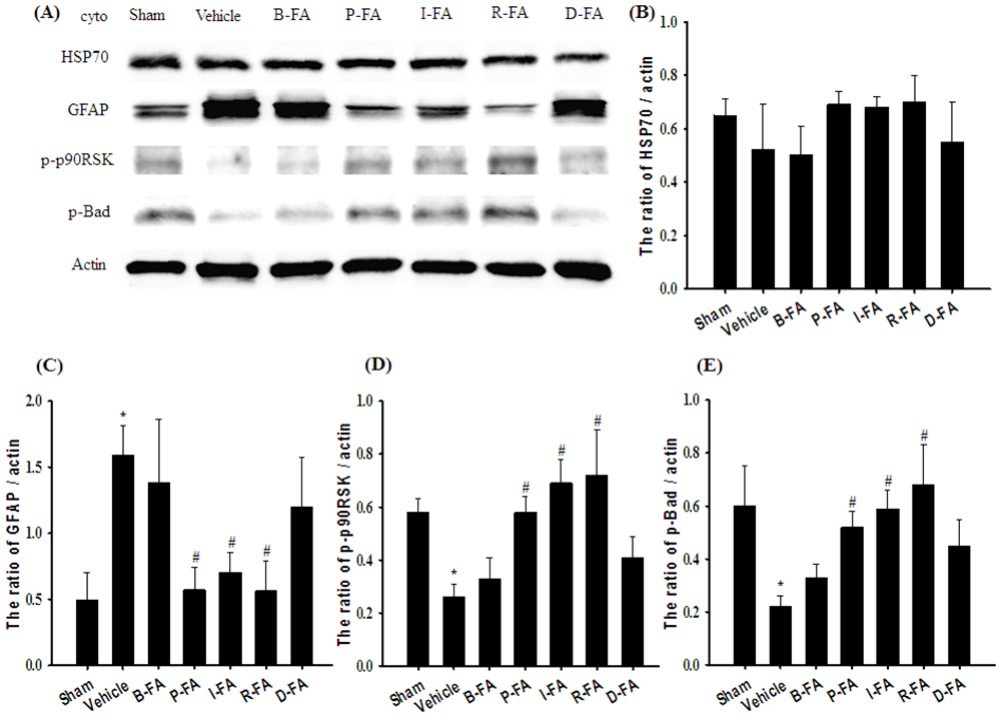
Effects of P-FA, I-FA, and R-FA on the cytosolic expression of HSP70, GFAP, p-p90RSK, and p-Bad in the cortical penumbra. (A) Representative Western blot images show cytosolic HSP70, GFAP, p-p90RSK, and p-Bad expression in the cortical penumbra in the Sham, Vehicle, B-FA, P-FA, I-FA, R-FA, and D-FA groups 7 d after reperfusion. Actin was used as a loading control in Western blot analysis. The ratios of (B) HSP70/actin, (C) GFAP/actin, (D) p-p90RSK/actin, and (E) p-Bad/actin expression were calculated in the cortical penumbra in the Sham, Vehicle, B-FA, P-FA, I-FA, R-FA, and D-FA groups (n = 4–5). Data are presented as mean ± SD. *P < 0.05 versus the Sham group; #*P* < 0.05 versus the Vehicle group.

The cytosolic expression of HSP70 in the cortical penumbra among the experimental groups showed nonsignificant differences 7 d after reperfusion (*P* > 0.05; Fig 4A and 4B). Cytosolic GFAP expression was markedly higher in the Vehicle group (3.2-fold) than in the Sham group (*P* < 0.05), and markedly lower in the P-FA (0.4-fold), I-FA (0.4-fold), and R-FA (0.4-fold) groups than in the Vehicle group (all *P* < 0.05; Fig 4A and 4C). By contrast, cytosolic p-p90RSK and p-Bad expression was significantly lower in the Vehicle group (0.4-fold and 0.4-fold, respectively) than in the Sham group (both *P* < 0.05), and significantly higher in the P-FA (2.2-fold and 2.4-fold, respectively), I-FA (2.7-fold and 2.7-fold, respectively), and R-FA (2.8-fold and 3.1-fold, respectively) groups than in the Vehicle groups (all *P* < 0.05; Fig 4A, 4D and 4E). Cytosolic GFAP, p-p90RSK, and p-Bad expression in the Vehicle, B-FA, and D-FA groups showed nonsignificant differences (*P* > 0.05).

### Effects of P-FA, I-FA, and R-FA on the Cytosolic Expression of p-CREB/CREB, Antiapoptotic (Bcl-2 and Bcl-xL), and Proapoptotic (Bax) Proteins

**Fig 5:**
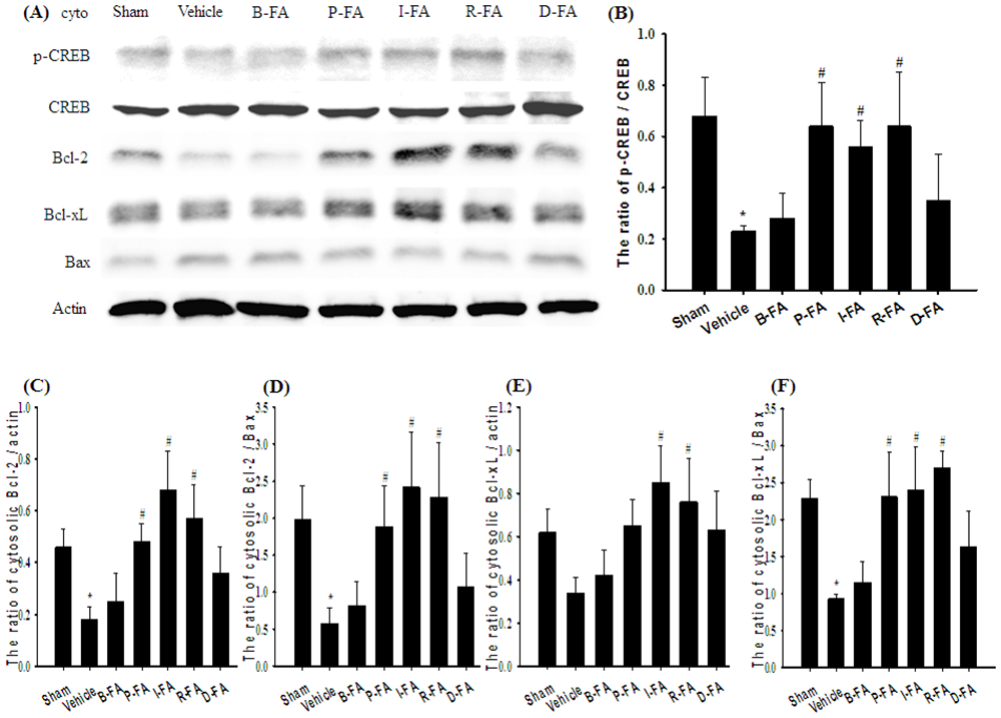
Effects of P-FA, I-FA, and R-FA on the cytosolic expression of p-CREB, CREB, Bcl-2, Bcl-xL, and Bax in the cortical penumbra. (A) Representative Western blot images show cytosolic p-CREB, CREB, Bcl-2, Bcl-xL, and Bax expression in the cortical penumbra in the Sham, Vehicle, B-FA, P-FA, I-FA, R-FA, and D-FA groups 7 d after reperfusion. The ratios of (B) p-CREB/CREB, (C) Bcl-2/actin, (D) Bcl-2/Bax, (E) Bcl-xL/actin, and (F) Bcl-xL/Bax expression were calculated in the cortical penumbra in the Sham, Vehicle, B-FA, P-FA, I-FA, R-FA, and D-FA groups (n = 4–5). Data are presented as mean ± SD. *P < 0.05 versus the Sham group; #*P* < 0.05 versus the Vehicle group.

The ratio of cytosolic p-CREB/CREB expression in the cortical penumbra was significantly lower in the Vehicle group (0.3-fold) than in the Sham group (*P* < 0.05), and significantly higher in the P-FA (2.8-fold), I-FA (2.4-fold), and R-FA (2.8-fold) groups than in the Vehicle group 7 d after reperfusion (all *P* < 0.05; Fig 5A and 5B). The ratio of cytosolic p-CREB/CREB expression in the Vehicle, B-FA, and D-FA groups showed nonsignificant differences (*P* > 0.05). Cytosolic Bcl-2 expression was significantly lower in the Vehicle group (0.4-fold) than in the Sham group (*P* < 0.05), and significantly higher in the P-FA (2.7-fold), I-FA (3.8-fold), and R-FA (3.2-fold) groups than in the Vehicle group (all *P* < 0.05; Fig 5A and 5C). By contrast, cytosolic Bcl-2 expression in the Vehicle, B-FA, and D-FA groups showed nonsignificant differences (*P* > 0.05). Cytosolic Bcl-xL expression was significantly higher in the I-FA (2.5-fold) and R-FA (2.2-fold) groups than in the Vehicle group (both *P* < 0.05; Fig 5A and 5E). However, cytosolic Bcl-xL expression in the Sham, Vehicle, B-FA, P-FA, and D-FA groups showed nonsignificant differences (*P* > 0.05). The ratios of cytosolic Bcl-2/Bax and Bcl-xL/Bax expression were significant lower in the Vehicle group (0.3-fold and 0.4-fold, respectively) than in the Sham group (both *P* < 0.05), and significantly higher in the P-FA (3.2-fold and 2.5-fold, respectively), I-FA (4.2-fold and 2.6-fold, respectively), and R-FA (3.9-fold and 2.9-fold, respectively) groups than in the Vehicle group (all *P* < 0.05; Fig 5A, 5D and 5F). The ratios of cytosolic Bcl-2/Bax and Bcl-xL/Bax expression in the Vehicle, B-FA, and D-FA groups showed nonsignificant differences (*P* > 0.05).

### Effects of P-FA, I-FA, and R-FA on the Mitochondrial Expression of Bcl-2, Bcl-xL, Bax, and AIF, and Cytosolic Expression of Cleaved Caspase-3 and AIF

**Fig 6:**
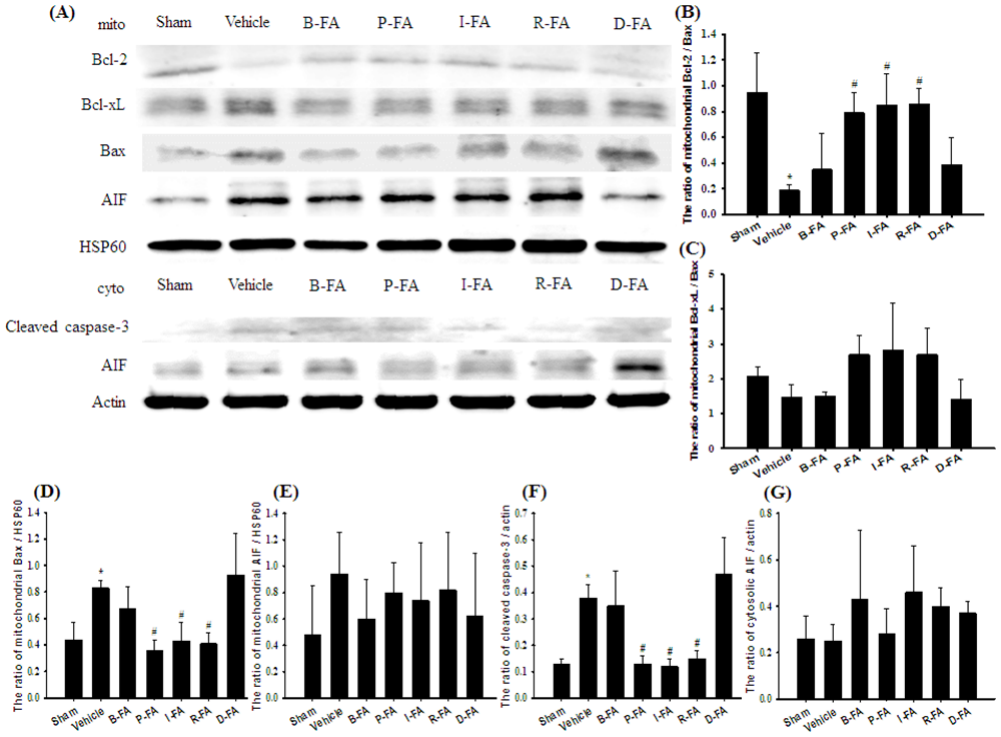
Effects of P-FA, I-FA, and R-FA on the mitochondrial expression of Bcl-2, Bcl-xL, AIF, and cytosolic expression of cleaved caspase-3 and AIF in the cortical penumbra. (A) Representative Western blot images show mitochondrial Bcl-2, Bcl-xL, and AIF expression, and cytosolic cleaved caspase-3 and AIF expression in the cortical penumbra in the Sham, Vehicle, B-FA, P-FA, I-FA, R-FA, and D-FA groups 7 d after reperfusion. HSP60 was used as a loading control in Western blot analysis. The ratios of (B) mitochondrial Bcl-2/Bax, (C) mitochondrial Bcl-xL/Bax, (D) mitochondrial Bax/HSP60, (E) mitochondrial AIF/HSP60, (F) cleaved caspase-3/actin, and (G) cytosolic AIF/actin expression were calculated in the cortical penumbra in the Sham, Vehicle, B-FA, P-FA, I-FA, R-FA, and D-FA groups (n = 4–5). mito, mitochondrial fraction. Data are presented as mean ± SD. *P < 0.05 versus the Sham group; #*P* < 0.05 versus the Vehicle group.

Mitochondrial Bax expression in the ischemic cortical penumbra was significantly higher in the Vehicle group (1.9-fold) than in the Sham group (*P* < 0.05), and significantly lower in the P-FA (0.4-fold), I-FA (0.5-fold), and R-FA (0.5-fold) groups than in the Vehicle group 7 d after reperfusion (all *P* < 0.05; Fig 6A and 6D). However, mitochondrial Bax expression in the Vehicle, B-FA, and D-FA groups showed nonsignificant differences (*P* > 0.05). The ratio of mitochondrial Bcl-2/Bax expression was significantly lower in the Vehicle group (0.2-fold) than in the Sham group (*P* < 0.05), and significantly higher in the P-FA (4.2-fold), I-FA (4.5-fold), and R-FA (4.5-fold) groups than in the Vehicle group (all *P* < 0.05; Fig 6A and 6B). The ratio of mitochondrial Bcl-2/Bax expression in the Vehicle, B-FA, and D-FA groups showed nonsignificant differences (*P* > 0.05). The ratio of mitochondrial Bcl-xL/Bax expression among the experimental groups showed nonsignificant differences (*P* > 0.05; Fig 6A and 6C). Cytosolic cleaved caspase-3 expression was significantly higher in the Vehicle group (2.9-fold) than in the Sham group (*P* < 0.05), and significantly lower in the P-FA (0.3-fold), I-FA (0.3-fold), and R-FA (0.4-fold) groups than in the Vehicle group (all *P* < 0.05; Fig 6A and 6F). Cytosolic cleaved caspase-3 expression in the Vehicle, B-FA, and D-FA groups showed nonsignificant differences (*P* > 0.05). Mitochondrial and cytosolic AIF expression among the experimental groups showed nonsignificant differences (*P* > 0.05; Fig 6A, 6E and 6G).

### Effects of P-FA, I-FA, and R-FA on the Expression of Cytochrome C-and Cleaved Caspase-3-Positive Cells

**Fig 7:**
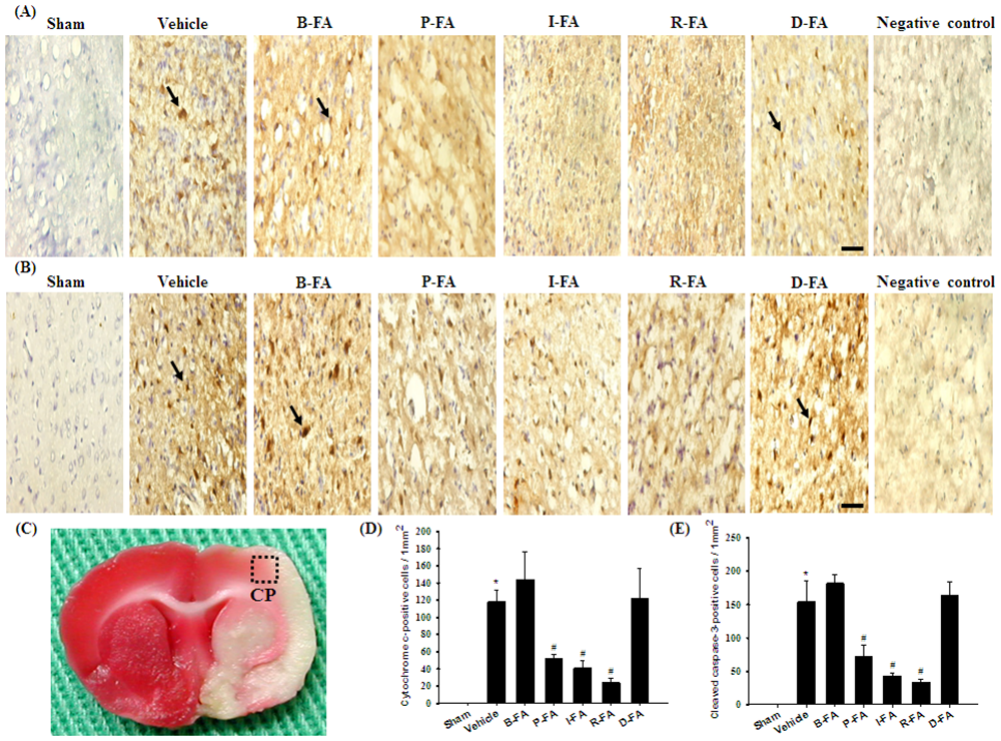
Effects of P-FA, I-FA, and R-FA on the expression of cytochrome c and cleaved caspase-3 in the cortical penumbra. Representative photographs show (A) cytochrome c and (B) cleaved caspase-3 expression in the cortical penumbra in the Sham, Vehicle, B-FA, P-FA, I-FA, R-FA, and D-FA groups 7 d after reperfusion. (C) Representative photograph shows TTC-stained rat brain coronal section. The dotted line square indicates the region of evaluation of immunopositive cells. CP, cortical penumbra. Dotted line square = 1mm^2^. The bar graphs show the numbers of (D) cytochrome c- and (E) cleaved caspase-3-positive cells in the cortical penumbra in the Sham, Vehicle, B-FA, P-FA, I-FA, R-FA, and D-FA groups (n = 3). Data are presented as mean ± SD. *P < 0.05 versus the Sham group; #*P* < 0.05 versus the Vehicle group. Arrows in (A) and (B) point to cytochrome c- and cleaved caspase-3-positive cells, respectively. Scale bars equal 50 μm for (A) and (B).

We evaluated the cytochrome c- and cleaved caspase-3-positive cells within the dotted line-square in the ischemic cortical penumbra (counts/1 mm^2^; Fig 7C) 7 d after reperfusion. The numbers of cytochrome c- and cleaved caspase-3-positive cells were significantly higher in the Vehicle group than in the Sham group (both *P* < 0.05), and significantly lower in the P-FA, I-FA, and R-FA groups than in the Vehicle group (all *P* < 0.05; Fig 7A, 7B, 7D and 7E). However, the numbers of cytochrome c- and cleaved caspase-3-positive cells in the Vehicle, B-FA, and D-FA groups showed nonsignificant differences (*P* > 0.05).

### Effects of P-FA, I-FA, and R-FA on the Expression of p-CREB/DAPI Double-Labeled Cells

**Fig 8:**
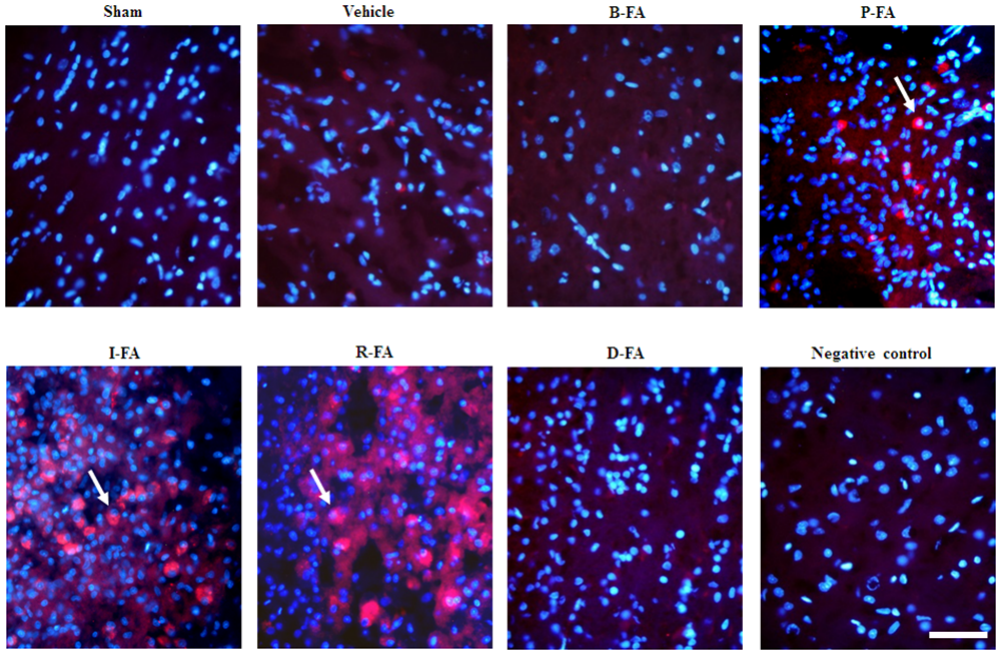
Effects of P-FA, I-FA, and R-FA on the expression of p-CREB/DAPI in the cortical penumbra. Representative photographs show p-CREB (red) colocalizing with DAPI (blue) in the cortical penumbra in the Sham, Vehicle, B-FA, P-FA, I-FA, R-FA, and D-FA groups 7 d after reperfusion. Arrows point to p-CREB/DAPI double-labeled cells. Scale bars = 5 μm.

We evaluated p-CREB/DAPI double-labeled cells within the dotted line-square in the ischemic cortical penumbra (Fig 7C). Analysis of p-CREB/DAPI costaining indicated strong cytoplasmic p-CREB immunoreactivity and intense nuclear p-CREB immunoreactivity in the cortical penumbra 7 d after reperfusion. P-CREB/DAPI double-labeled cell immunoreactivity was markedly higher in the P-FA, I-FA, and R-FA groups than in the Vehicle group (Fig 8). However, p-CREB/DAPI double-labeled cell immunoreactivity in the Vehicle, B-FA, and D-FA groups showed nonsignificant differences.

### Effects of D+I-FA and SB+I-FA on Cerebral Infarct Area

**Fig 9:**
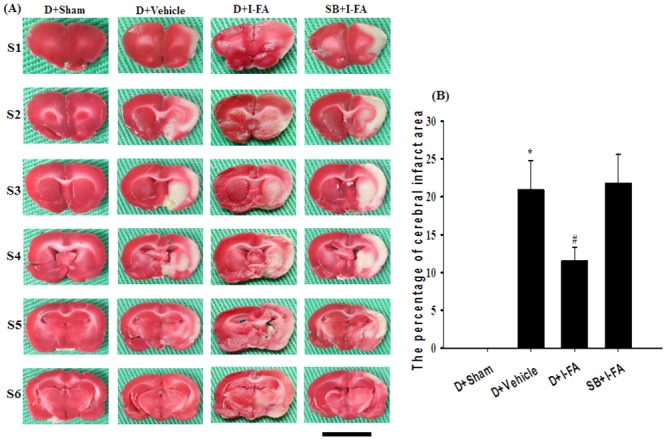
Cerebral infarction among the D+Sham, D+Vehicle, D+I-FA, and SB+I-FA groups after 30 min of MCAo followed by 7 d of reperfusion. (A) TTC staining shows normal brain tissues (S1–S6) (deep red) and infarct tissues (S1–S6) (white). Scale bar = 1 cm. (B) The percentage cerebral infarct areas in the D+Sham, D+Vehicle, D+I-FA, and SB+I-FA groups were evaluated 7 d after reperfusion (n = 3). Data are presented as mean ± SD. *P < 0.05 versus the Sham group; #*P* < 0.05 versus the Vehicle group.

In Experiment C, we evaluated the infarct areas in the D+Sham, D+Vehicle, D+I-FA, and SB+I-FA groups 7 d after reperfusion. The percentage cerebral infarct areas was significantly higher in the D+Vehicle group than in the D+Sham group (*P* < 0.05), and significantly lower in the D+I-FA group than in the D+Vehicle group (*P* < 0.05; Fig 9A and 9B). The tendency for cerebral infarction in the D+Sham, D+Vehicle, and D+I-FA groups was similar to that in the Sham, Vehicle, and I-FA groups in Experiment A. The percentage cerebral infarct areas in the D+Vehicle and SB+I-FA groups showed nonsignificant differences (*P* > 0.05).

### Effects of D+I-FA and SB+I-FA on the Cytosolic Expression of p-p38 MAPK/p38 MAPK, p-p90RSK, p-Bad, p-CREB/CREB, and Bcl-2/Bax

**Fig 10:**
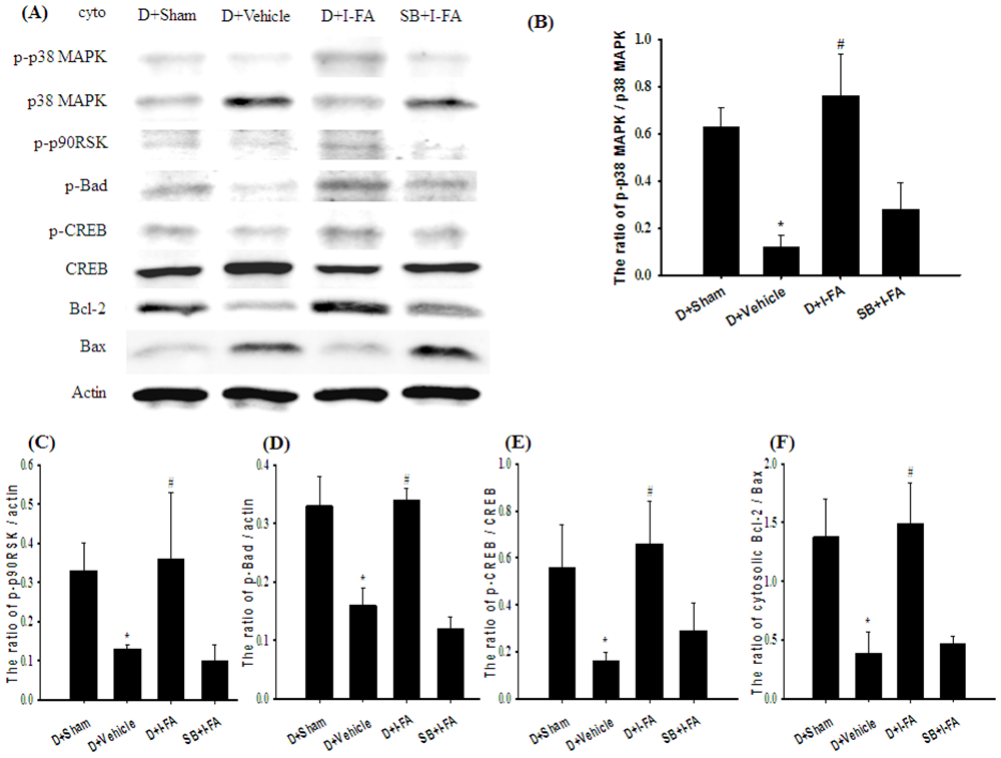
Effects of D+I-FA and SB+I-FA on the cytosolic expression of p-p38 MAPK, p38 MAPK, p-p90RSK, p-Bad, p-CREB, CREB, and Bcl-2/Bax. (A) Representative images show cytosolic p-p38 MAPK, p38 MAPK, p-p90RSK, p-Bad, p-CREB, CREB, Bcl-2, and Bax expression in the cortical penumbra in the D+Sham, D+Vehicle, D+I-FA, and SB+I-FA groups 7 d after reperfusion. The ratios of (B) p-p38 MAPK/p38 MAPK, (C) p-p90RSK/actin, (D) p-Bad/actin, (E) p-CREB/CREB, and (F) Bcl-2/Bax were calculated in the cortical penumbra in the D+Sham, D+Vehicle, D+I-FA, and SB+I-FA groups (n = 5). Data are presented as mean ± SD. *P < 0.05 versus the D+Sham group; #*P* < 0.05 versus the D+Vehicle group.

Cytosolic p-p38 MAPK/p38 MAPK, p-p90RSK, p-Bad, p-CREB/CREB, and Bcl-2/Bax expression in the cortical penumbra was significantly lower in the D+Vehicle group (0.2-fold, 0.4-fold, 0.5-fold, 0.3-fold, and 0.3-fold, respectively) than in the D+Sham group (all *P* < 0.05), and significantly higher in the D+I-FA group (6.3-fold, 2.8-fold, 2.0-fold, 4.1-fold, and 3.8-fold, respectively) than in the D+Vehicle group (all *P* < 0.05; Fig 10A, 10B, 10C, 10D, 10E and 10F, respectively). However, cytosolic p-p38 MAPK/p38 MAPK, p-p90RSK, p-Bad, p-CREB/CREB, and Bcl-2/Bax expression in the D+Vehicle and SB+I-FA groups showed nonsignificant differences (*P* > 0.05).

### Effects of D+I-FA and SB+I-FA on the Mitochondrial Expression of Bcl-2/Bax and Bax, and Cytosolic Expression of Cleaved Caspase-3

**Fig 11:**
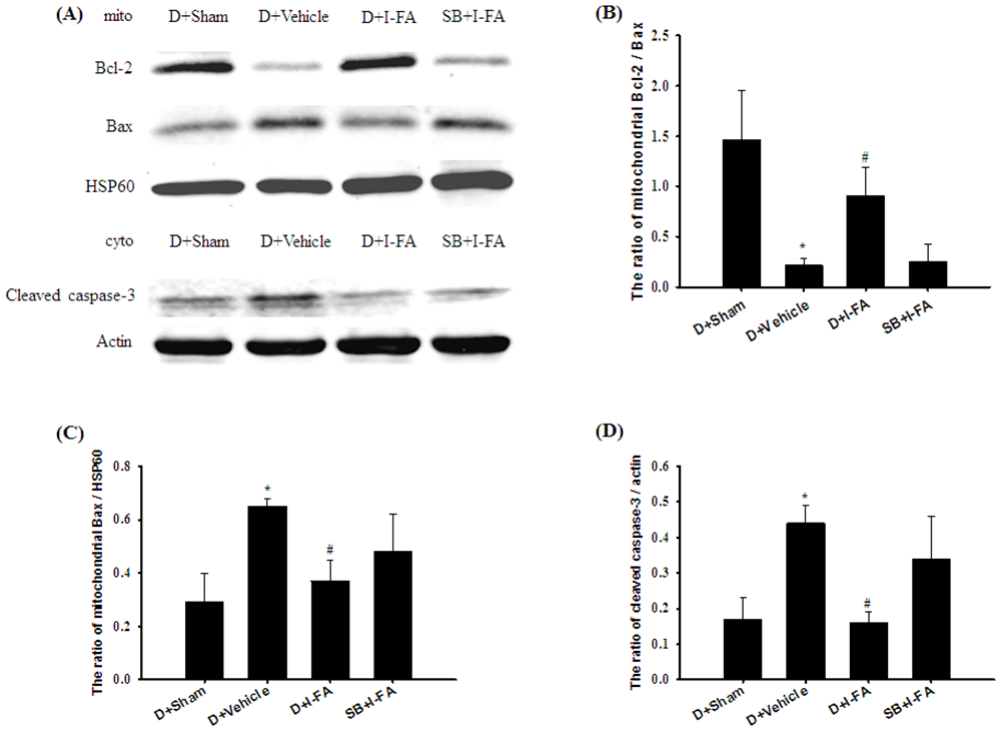
Effects of D+I-FA and SB+I-FA on the mitochondrial Bcl-2 and Bax, and cytosolic cleaved caspase-3 expression in the cortical penumbra. (A) Representative images show mitochondrial Bcl-2 and Bax, and cytosolic cleaved caspase-3 expression in the cortical penumbra in the D+Sham, D+Vehicle, D+I-FA, and SB+I-FA groups 7 d after reperfusion. The ratios of (B) mitochondrial Bcl-2/Bax, (C) mitochondrial Bax/HSP60, and (D) cleaved caspase-3/actin were calculated in the cortical penumbra in the D+Sham, D+Vehicle, D+I-FA, and SB+I-FA groups (n = 5). Data are presented as mean ± SD. *P < 0.05 versus the D+Sham group; #*P* < 0.05 versus the D+Vehicle group.

Mitochondrial Bax and cytosolic cleaved caspase-3 expression in the cortical penumbra was significantly higher in the D+Vehicle group (2.2-fold and 2.6-fold, respectively) than in the D+Sham group (both *P* < 0.05), and significantly lower in the D+I-FA group (0.6-fold and 0.4-fold, respectively) than in the D+Vehicle group (both *P* < 0.05; Fig 11A, 11C and 11D). The ratio of mitochondrial Bcl-2/Bax expression was significantly lower in the D+Vehicle group (0.2-fold) than in the D+Sham group (*P* < 0.05), and significantly higher in the D+I-FA group (4.1-fold) than in D+Vehicle group (*P* < 0.05; Fig 11A and 11B). However, mitochondrial Bcl-2/Bax and Bax expression, and cytosolic cleaved caspase-3 expression in the D+Vehicle and SB+I-FA groups showed nonsignificant differences (*P* > 0.05).

## Discussion

In this study, we evaluated cerebral infarction in a rat model of 30 min of MCAo followed by 7 d of reperfusion. Our results are in accordance with those from our previous study, which indicated that mild cerebral ischemia (30 min of MCAo) caused gross infarct expansion in the MCA territory involving the cortex and striatum in the subacute phase (7 d) of cerebral ischemia/reperfusion (I/R) injury [6]. Previous studies have shown that FA administered immediately after cerebral ischemia provides neuroprotective effects against cerebral infarction in the acute phase of transient [21] and permanent [23] MCAo. In this study, we first evaluated the anti-infarct effects of FA administered at various time points during the subacute phase of cerebral I/R injury. Our results indicated that FA administered 2 h before ischemia (P-FA), immediately after ischemia (I-FA), or 2 h after reperfusion (R-FA) significantly reduced cerebral infarct area and improved neurological behavior in rats subjected to 30 min of MCAo followed by 7 d of reperfusion, whereas FA administered 24 h before ischemia (B-FA) or 24 h after reperfusion (D-FA) failed to reduce infarct size or neurological deficits. Increasing evidence has indicated that reactive astrocytes accumulate in the periinfarct region and subsequently trigger reactive astrogliosis, which exacerbates brain damage and contributes to infarct expansion in the subacute phase of cerebral ischemia [29,30]. Thus, pharmacologic inhibition of astrocytic activation mitigates delayed infarct expansion during the subacute phase of MCAo [31]. Our immunoblotting results indicated that GFAP, a marker of astrocytes, was significantly upregulated in the cortical penumbra after MCAo. However, this increase in GFAP expression was effectively downregulated in the P-FA, I-FA, and R-FA groups 7 d after reperfusion. These results suggest that FA administered 2 h prior to cerebral ischemia, and up to 2 h after reperfusion, exerts neuroprotective effects against cerebral I/R injury, and that these effects are at least partially attributed to the downregulation of astrocyte-mediated infarct expansion in the cortical penumbra 7 d after reperfusion.

MAPKs, including JNK, ERK1/2, and p38 MAPK, are activated after focal cerebral ischemia and play a prominent role in the transduction of stress-induced signals by protein kinases, which regulate neuronal survival or damage [3,32]. Studies have indicated that MAPKs induce or mitigate apoptosis, a critical role in infarct expansion, by modulating various signaling pathways, and that astrocytic activation closely parallels the expression of proapoptotic factors in the periinfarct area during the subacute phase of cerebral ischemia [6,7,29]. JNK activation elicits apoptosis in response to ischemic stress, and JNK inhibitors provide neuroprotection after cerebral I/R injury [33]. By contrast, ERK1/2 regulates cell growth, proliferation, and apoptosis, and activation of ERK1/2 is generally associated with neuronal survival during cerebral ischemia [34]. p38 MAPK plays a dual role in the regulation of cell death. Studies have shown that activation of p38 MAPK promotes the production of proinflammatory cytokines and exacerbates infarction after focal cerebral ischemia [3,35], and that the phosphorylation of p38 MAPK exerts neuroprotective effects against cerebral ischemic injury by activating antiapoptotic signals in the ischemic cortex in hypoxic precondition [1] and transient MCAo [5,6] models. Pharmacologic activation of the PI3K/Akt signaling pathway exerts antiapoptotic effects in the ischemic penumbra after cerebral I/R injury [36]. Therefore, intervention in the MAPK or PI3K/Akt signaling cascade is critical for cell survival and reduction of cerebral infarction following ischemic injury. Our immunoblotting results indicated that p-p38 MAPK was derived from total p38 MAPK, and that the ratio of p-p38 MAPK/p38 MAPK, but not p-JNK/JNK, p-ERK1/2/ERK1/2, or p-Akt/Akt, expression, was significantly downregulated in the cytosolic fraction in the cortical penumbra after MCAo, but this expression was effectively restored in the P-FA, I-FA, and R-FA groups 7 d after reperfusion. These results suggest that P-FA, I-FA, and R-FA effectively activate p38 MAPK signaling, and that the effects of FA treatment on astrocyte-mediated infarct expansion are most likely attributed to the modulation of p38-, but not JNK-, ERK1/2-, or Akt-, induced antiapoptotic signaling in the cortical penumbra 7 d after reperfusion.

Accumulating evidence has indicated that activated ERK1/2 phosphorylates p90RSK and subsequently triggers the phosphorylation of Bad, resulting in neuroprotection against focal cerebral I/R injury [7]. Considerable cross-talk and interaction occur between ERK1/2 and p38 MAPK signaling pathways in response to mitogenic or stress stimuli [9,37,38]. Lian et al. (1999) demonstrated that a pharmacologic inhibitor of p38 MAPK signaling effectively blocks the activation of ERK1/2 and p90RSK in stimulated neutrophils, indicating a close interaction between p90RSK and p38 MAPK signaling [11]. During cerebral ischemia, p-p90RSK phosphorylates the proapoptotic protein Bad. P-Bad subsequently binds to 14-3-3, which prevents Bad interaction with Bcl-xL (Bcl-2) and inhibits proapoptotic protein Bax translocation to the mitochondria in the ischemic cortex after MCAo [7,39]. ERK1/2/p90RSK and p38 MAPK-activated kinases have major roles in the activation of CREB in the periinfarct area after focal cerebral ischemia [40,41]. CREB is a transcription factor and plays neuroprotective roles in the regulation of various cellular responses, such as synaptic plasticity, proliferation, and survival in many cell types [42]. Previous studies have shown that CREB phosphorylation at serine 133, and subsequent CRE-mediated gene transcription of antiapoptotic factors, including Bcl-2 and Bcl-xL, occurs in the penumbral cortex or hippocampus during various stages of focal cerebral ischemia [6,14,42]. HSP70 is also a downstream target of p38 MAPK and plays a role in the downregulation of proapoptotic factors in in vitro [43] and in vivo [44] models. We observed that cytosolic p-p90RSK and p-Bad expression were markedly downregulated in the cortical penumbra after MCAo; but P-FA, I-FA, and R-FA effectively restored the cytosolic expression of these proteins 7 d after reperfusion. By contrast, FA treatment did not alter the cytosolic expression of HSP70 in the cortical penumbra. The ratio of p-CREB/CREB expression was markedly decreased in the cytosolic fraction in the cortical penumbra after MCAo, but the ratio increased after P-FA, I-FA, and R-FA treatment. Further analysis of p-CREB/DAPI costaining indicated that the pattern of p-CREB immunofluorescent costaining in the cortical penumbra of the FA treatment groups was similar to that shown by p-CREB immunoblotting, and that p-CREB immunoreactivity in the cytoplasma closely paralleled that in the nuclear compartment in the cortical penumbra. These results are in accordance with those from a previous study, which showed that p-CREB expression in the cytosol is positively related to that in the nucleus, and reflects the activation and nuclear translocation of CREB after hypoxic preconditioning [45]. Therefore, our result suggest that P-FA, I-FA, and R-FA effectively activate p90RSK/Bad- and CREB-mediated survival signaling, and that the survival-promoting effects of FA treatment are possibly attributed to the upregulation of p38 MAPK/p90RSK/CREB-, but not p38 MAPK/HSP70-, mediated anti-apoptotic signaling in the cortical penumbra 7 d after reperfusion.

The Bcl-2 protein family is classified into three structurally distinct subgroups: antiapoptotic proteins such as Bcl-2 and Bcl-xL, proapoptotic proteins such as Bax, and Bcl-2 homology 3 (BH3)-only proteins such as Bad [46,47]. The dynamic balance between antiapoptotic Bcl-2 (Bcl-xL) and proapoptotic Bax proteins plays a key role in determining cell fate during cerebral ischemia [48]. Accumulating evidence has indicated that an increase in the Bcl-2 (Bcl-xL)/Bax ratio inhibits Bax translocation to the mitochondria and protects neurons against apoptotic insults, whereas a shift in the balance toward an excess of Bax elicits ischemia-induced neuronal apoptosis [16,49,50]. In response to apoptotic stimuli, Bax dissociates from Bcl-2/Bax heterodimers in the cytosol. Monomeric Bax then translocates to the mitochondria where it can be cross-linked to form homo-oligomers and consequently permeabilize the mitochondrial outer membrane, resulting in cytochrome c release [51]. In the intrinsic apoptotic pathway, cytochrome c binds to and activates apoptotic protease activating factor-1 and procaspase-9 to form an apoptosome, and then initiates caspase-3-mediated apoptosis [52]. Previous studies have shown that Bax-mediated mitochondrial release of AIF predominantly expressed in the ischemic cortex or hippocampus translocates to the nucleus and undergoes caspase-independent apoptosis 8–72 h after cerebral I/R injury, whereas overexpression of Bcl-2 (Bcl-xL) effectively inhibits the AIF-mediated cell death pathway [53,54]. Our study results indicated that cytosolic Bcl-2 expression was significantly downregulated in the cortical penumbra after MCAo, but P-FA, I-FA, and R-FA effectively restored cytosolic Bcl-2 levels 7 d after reperfusion, and I-FA and R-FA also upregulated cytosolic Bcl-xL expression. Further analysis revealed that the ratios of cytosolic and mitochondrial Bcl-2/Bax, and of cytosolic Bcl-xL/Bax, were lower in the cortical penumbra after MCAo, but P-FA, I-FA, and R-FA effectively restored these ratios. By contrast, the mitochondrial expression of Bax, cytochrome c (IHC results), and cleaved caspase-3 (immunoblotting and IHC results), were markedly increased in the cortical penumbra after MCAo, and P-FA, I-FA, and R-FA effectively downregulated the expression of these proteins 7 d after reperfusion. FA treatment did not influence the mitochondrial expression of Bcl-xL, or cytosolic or mitochondrial expression of AIF. The results indicate that FA treatment at our 3 evaluated time points actively inhibits Bax mitochondrial translocation by upregulating cytosolic Bcl-2 and Bcl-xL, and that the inhibiting effects of FA treatment on Bax translocation contribute to the downregulation of mitochondrial Bax expression, upregulation of the Bcl-2/Bax (but not Bcl-xL/Bax) ratio, and consequent inhibition of cytochrome c-mediated caspase-3 activation. According to these results, we propose that p38 MAPK/p90RSK/CREB/Bcl-2-related antiapoptotic signaling might be involved in the neuroprotective effects of P-FA, I-FA, and R-FA, and that the effects of FA treatment on Bax-mediated apoptosis can be further attributed to the downregulation of cytochrome c/caspase-3-mediated, but not AIF-mediated, apoptotic signaling in the cortical penumbra 7 d after reperfusion.

To evaluate the possible role of p38 MAPK in attenuating Bax-mediated apoptosis in the I-FA group, the representative group of FA treatment, we designed another experiment to investigate the action of SB203580, a specific inhibitor of the p38 MAPK pathway. We observed that a DMSO (vehicle control) pretreatment (D+I-FA) did not modify the effects of I-FA on cerebral infarct size or modulation of p38 MAPK-related proteins. However, a SB203580 pretreatment (SB+I-FA) fully abrogated the upregulating effects of I-FA on p-p38 MAPK expression. SB+I-FA consequently suppressed p90RSK/Bad/CREB/Bcl-2 signaling and triggered the Bax-mediated caspase-3-dependent apoptotic pathway in the cortical penumbra, leading to increased cerebral infarction 7 d after reperfusion. Based on these results, we propose that P-FA, I-FA, and R-FA protect against cerebral infarction by activating p38 MAPK signaling, and that the attenuating effects of FA treatment on Bax-mediated apoptosis are caused by the upregulation of the p38 MAPK/p90RSK/CREB/Bcl-2 anti-apoptotic pathway in the cortical penumbra 7 d after reperfusion. According to our research, our study is the first to show that FA is neuroprotective against Bax-mediated apoptosis by regulating p38 MAPK-, but not ERK1/2-, mediated p90RSK/CREB/Bcl-2 signaling in the subacute phase of cerebral I/R injury.

## Conclusions

Overall, our study results suggest that FA administered during the period between 2 h before ischemia and 2 h after reperfusion effectively reduces cerebral infarction 7 d after reperfusion, and that the anti-infarct effects of FA involve the suppression of reactive astrocytosis, which closely parallels the upregulation of proapoptotic factors in the cortical penumbra. The effects of FA treatment on Bax-mediated apoptosis can be attributed to the activation of p38 MAPK/p90RSK/CREB/Bcl-2 signaling, which maintains mitochondrial outer membrane integrity and inhibits the cytochrome c-mediated caspase-3 apoptotic pathway in the cortical penumbra 7 d after reperfusion. FA is known to exert antiinflammatory, antioxidative, and antiapoptotic effects in the acute phase of cerebral ischemia. Our study results further indicate that pre- and posttreatment FA elicits neuroprotection against infarct expansion in the subacute phase after cerebral I/R injury. Therefore, FA provides a potential strategy for stroke prevention and a promising therapeutic strategy in the subacute phase after cerebral ischemia. However, further investigation is required to fully characterize MAPK-mediated signaling and extend the therapeutic time window of FA for future clinical application.

## Supporting Information

S1 Table Data collection for cerebral infarct evaluation, neurological examination, western blot analysis, and immunohistochemical assessment. (DOC)
