# Supplementary material for: Ferulic Acid Administered at Various Time Points Protects against Cerebral Infarction by Activating p38 MAPK/p90RSK/CREB/Bcl-2 Anti-Apoptotic Signaling in the Subacute Phase of Cerebral Ischemia-Reperfusion Injury in Rats
Source: PLoS One. 2016 May 17;11(5):e0155748. doi: 10.1371/journal.pone.0155748 (PMC4871485; doi:10.1371/journal.pone.0155748)
Supplement: S1 Table — (DOC) [file pone.0155748.s001.doc]

**Data collection for cerebral infarct evaluation, neurological examination, western blot analysis, and immunohistochemical assessment.**

1. The percentage of infarct area was calculated at 7 d of reperfusion

|  | Sham | vehicle | B-FA | P-FA | I-FA | R-FA | D-FA |
| --- | --- | --- | --- | --- | --- | --- | --- |
| 1 | 0 | 20.0 | 29.1 | 11.2 | 10.9 | 8.5 | 15.9 |
| 2 | 0 | 21.4 | 27.1 | 10.2 | 13.4 | 10.3 | 20.9 |
| 3 | 0 | 27.7 | 25.8 | 20.7 | 11.3 | 10.8 | 22.4 |
| 4 | 0 | 25.1 | 21.7 | 11.4 | 15.1 | 7.9 | 22.9 |
| 5 | 0 | 26.4 | 20.3 | 13.4 | 8.3 | 15.2 | 19.1 |
| 6 | 0 | 24.1 | 24.9 | 10.5 | 11.7 | 11.9 | 20.5 |
| **Mean** | **0** | **24.1** | **24.8** | **12.9** | **11.8** | **10.8** | **20.3** |
| **SD** | **0** | **2.9** | **3.3** | **4.0** | **2.3** | **2.6** | **2.5** |

2. The neurological deficit scores were measured at 1, 3 and 7 d of reperfusion

(1) One day of reperfusion

|  | Sham | Vehicle | B-FA | P-FA | I-FA | R-FA | D-FA |
| --- | --- | --- | --- | --- | --- | --- | --- |
| 1 | 0 | 7 | 8 | 5 | 5 | 6 | 7 |
| 2 | 0 | 7 | 6 | 6 | 5 | 5 | 7 |
| 3 | 0 | 8 | 7 | 7 | 6 | 6 | 7 |
| 4 | 0 | 7 | 8 | 6 | 6 | 5 | 8 |
| 5 | 0 | 7 | 7 | 7 | 6 | 6 | 7 |
| 6 | 0 | 7 | 7 | 5 | 5 | 6 | 7 |
| **Mean** | **0** | **7.2** | **7.2** | **6.0** | **5.5** | **5.7** | **7.2** |
| **SD** | **0** | **0.4** | **0.8** | **0.9** | **0.5** | **0.5** | **0.4** |

(2) Three days of reperfusion

|  | Sham | Vehicle | B-FA | P-FA | I-FA | R-FA | D-FA |
| --- | --- | --- | --- | --- | --- | --- | --- |
| 1 | 0 | 7 | 6 | 5 | 5 | 6 | 7 |
| 2 | 0 | 7 | 7 | 6 | 5 | 5 | 6 |
| 3 | 0 | 8 | 7 | 7 | 6 | 5 | 7 |
| 4 | 0 | 8 | 7 | 6 | 6 | 5 | 6 |
| 5 | 0 | 7 | 7 | 6 | 6 | 6 | 6 |
| 6 | 0 | 7 | 8 | 5 | 5 | 6 | 7 |
| **Mean** | **0** | **7.3** | **7.0** | **5.8** | **5.5** | **5.5** | **6.5** |
| **SD** | **0** | **0.5** | **0.6** | **0.8** | **0.5** | **0.5** | **0.5** |

(3) Seven days of reperfusion

|  | Sham | Vehicle | B-FA | P-FA | I-FA | R-FA | D-FA |
| --- | --- | --- | --- | --- | --- | --- | --- |
| 1 | 0 | 7 | 6 | 4 | 5 | 5 | 5 |
| 2 | 0 | 7 | 7 | 5 | 4 | 5 | 6 |
| 3 | 0 | 8 | 8 | 6 | 5 | 4 | 7 |
| 4 | 0 | 7 | 7 | 5 | 5 | 4 | 6 |
| 5 | 0 | 9 | 8 | 5 | 5 | 6 | 6 |
| 6 | 0 | 7 | 9 | 5 | 5 | 5 | 7 |
| **Mean** | **0** | **7.5** | **7.5** | **5.0** | **4.8** | **4.8** | **6.2** |
| **SD** | **0** | **0.8** | **1.0** | **0.6** | **0.4** | **0.8** | **0.8** |

Figure 3

Immunoblotting

1. The ratios of p-JNK/JNK in the cortical penumbra among the experimental groups at 7 d of reperfusion

|  | Sham | Vehicle | B-FA | P-FA | I-FA | R-FA | D-FA |
| --- | --- | --- | --- | --- | --- | --- | --- |
| 1 | 0.22 | 0.30 | 0.22 | 0.16 | 0.22 | 0.24 | 0.56 |
| 2 | 0.29 | 0.35 | 0.39 | 0.32 | 0.22 | 0.27 | 0.36 |
| 3 | 0.27 | 0.50 | 0.30 | 0.45 | 0.35 | 0.30 | 0.26 |
| 4 | 0.50 | 0.25 | 0.30 | 0.14 | 0.33 | 0.16 | 0.18 |
| **5** | 0.34 | 0.55 | 0.56 | 0.38 | 0.38 | 0.42 | 0.27 |
| **Mean** | **0.32** | **0.39** | **0.35** | **0.29** | **0.30** | **0.28** | **0.33** |
| **SD** | **0.11** | **0.13** | **0.13** | **0.14** | **0.08** | **0.09** | **0.15** |

2. The ratios of p-ERK/ERK in the cortical penumbra among the experimental groups at 7 d of reperfusion

|  | Sham | Vehicle | B-FA | P-FA | I-FA | R-FA | D-FA |
| --- | --- | --- | --- | --- | --- | --- | --- |
| 1 | 0.33 | 0.40 | 0.62 | 0.26 | 0.38 | 0.43 | 0.87 |
| 2 | 0.27 | 0.50 | 0.84 | 0.49 | 0.43 | 0.29 | 0.34 |
| 3 | 0.14 | 0.96 | 0.33 | 0.73 | 0.24 | 0.59 | 0.25 |
| 4 | 0.53 | 0.41 | 0.50 | 0.25 | 0.46 | 0.28 | 0.31 |
| **5** | 0.40 | 0.32 | 0.66 | 0.59 | 0.48 | 0.50 | 0.47 |
| **Mean** | **0.33** | **0.52** | **0.59** | **0.46** | **0.40** | **0.42** | **0.45** |
| **SD** | **0.15** | **0.26** | **0.19** | **0.21** | **0.10** | **0.13** | **0.25** |

3. The ratios of p-p38/p38 MAPK in the cortical penumbra among the experimental groups at 7 d of reperfusion

|  | Sham | Vehicle | B-FA | P-FA | I-FA | R-FA | D-FA |
| --- | --- | --- | --- | --- | --- | --- | --- |
| 1 | 1.23 | 0.41 | 0.36 | 1.16 | 1.94 | 1.36 | 0.54 |
| 2 | 1.66 | 0.44 | 0.60 | 1.29 | 1.35 | 1.34 | 0.49 |
| 3 | 1.77 | 0.68 | 0.58 | 1.07 | 1.02 | 1.75 | 1.20 |
| 4 | 1.72 | 0.60 | 0.73 | 1.85 | 1.20 | 1.71 | 0.97 |
| **5** | 1.25 | 0.45 | 1.06 | 1.19 | 1.56 | 1.38 | 0.85 |
| **Mean** | **1.53** | **0.52** | **0.67** | **1.31** | **1.41** | **1.51** | **0.81** |
| **SD** | **0.12** | **0.26** | **0.26** | **0.31** | **0.35** | **0.20** | **0.30** |

4. The ratios of p-Akt/Akt in the cortical penumbra among the experimental groups at 7 d of reperfusion

|  | Sham | Vehicle | B-FA | P-FA | I-FA | R-FA | D-FA |
| --- | --- | --- | --- | --- | --- | --- | --- |
| 1 | 0.25 | 0.37 | 0.16 | 0.47 | 0.41 | 0.51 | 0.81 |
| 2 | 0.20 | 0.33 | 0.59 | 0.65 | 0.50 | 0.52 | 0.56 |
| 3 | 0.41 | 0.51 | 0.69 | 0.74 | 0.80 | 0.36 | 0.66 |
| 4 | 0.35 | 0.74 | 0.84 | 0.56 | 0.64 | 0.48 | 0.37 |
| **Mean** | **0.30** | **0.49** | **0.57** | **0.61** | **0.59** | **0.47** | **0.60** |
| **SD** | **0.10** | **0.19** | **0.29** | **0.12** | **0.17** | **0.07** | **0.18** |

Figure 4

Immunoblotting

1. The ratios of HSP70/actin in the cortical penumbra among the experimental groups at 7 d of reperfusion

|  | Sham | Vehicle | B-FA | P-FA | I-FA | R-FA | D-FA |
| --- | --- | --- | --- | --- | --- | --- | --- |
| 1 | 0.62 | 0.71 | 0.52 | 0.76 | 0.72 | 0.70 | 0.67 |
| 2 | 0.65 | 0.48 | 0.35 | 0.67 | 0.67 | 0.76 | 0.34 |
| 3 | 0.59 | 0.31 | 0.50 | 0.66 | 0.63 | 0.78 | 0.66 |
| 4 | 0.74 | 0.56 | 0.61 | 0.66 | 0.69 | 0.55 | 0.52 |
| **Mean** | **0.65** | **0.52** | **0.50** | **0.69** | **0.68** | **0.70** | **0.55** |
| **SD** | **0.17** | **0.17** | **0.11** | **0.05** | **0.04** | **0.10** | **0.15** |

2. The ratios of GFAP/actin in the cortical penumbra among the experimental groups at 7 d of reperfusion

|  | Sham | Vehicle | B-FA | P-FA | I-FA | R-FA | D-FA |
| --- | --- | --- | --- | --- | --- | --- | --- |
| 1 | 0.61 | 1.54 | 2.24 | 0.47 | 0.80 | 0.42 | 1.19 |
| 2 | 0.57 | 1.77 | 1.14 | 0.80 | 0.58 | 0.65 | 1.34 |
| 3 | 0.45 | 1.30 | 1.19 | 0.64 | 0.90 | 0.89 | 1.28 |
| 4 | 0.67 | 1.49 | 1.26 | 0.56 | 0.67 | 0.29 | 1.60 |
| **5** | 0.14 | 1.84 | 1.08 | 0.36 | 0.54 | 0.55 | 0.60 |
| **Mean** | **0.49** | **1.59** | **1.38** | **0.57** | **0.70** | **0.56** | **1.20** |
| **SD** | **0.21** | **0.22** | **0.48** | **0.17** | **0.15** | **0.23** | **0.37** |

3. The ratios of p-p90RSK/actin in the cortical penumbra among the experimental groups at 7 d of reperfusion

|  | Sham | Vehicle | B-FA | P-FA | I-FA | R-FA | D-FA |
| --- | --- | --- | --- | --- | --- | --- | --- |
| 1 | 0.67 | 0.31 | 0.37 | 0.58 | 0.81 | 0.98 | 0.53 |
| 2 | 0.54 | 0.24 | 0.24 | 0.55 | 0.63 | 0.64 | 0.37 |
| 3 | 0.57 | 0.30 | 0.31 | 0.55 | 0.58 | 0.80 | 0.42 |
| 4 | 0.57 | 0.25 | 0.44 | 0.69 | 0.73 | 0.63 | 0.42 |
| **5** | 0.54 | 0.18 | 0.27 | 0.53 | 0.72 | 0.55 | 0.31 |
| **Mean** | **0.58** | **0.26** | **0.33** | **0.58** | **0.69** | **0.72** | **0.41** |
| **SD** | **0.05** | **0.05** | **0.08** | **0.06** | **0.09** | **0.17** | **0.08** |

4. The ratios of p-Bad/actin in the cortical penumbra among the experimental groups at 7 d of reperfusion

|  | Sham | Vehicle | B-FA | P-FA | I-FA | R-FA | D-FA |
| --- | --- | --- | --- | --- | --- | --- | --- |
| 1 | 0.55 | 0.21 | 0.26 | 0.53 | 0.62 | 0.69 | 0.37 |
| 2 | 0.54 | 0.20 | 0.33 | 0.56 | 0.49 | 0.63 | 0.43 |
| 3 | 0.47 | 0.27 | 0.34 | 0.58 | 0.57 | 0.90 | 0.42 |
| 4 | 0.59 | 0.25 | 0.40 | 0.43 | 0.67 | 0.67 | 0.39 |
| **5** | 0.86 | 0.18 | 0.30 | 0.50 | 0.59 | 0.49 | 0.62 |
| **Mean** | **0.60** | **0.22** | **0.33** | **0.52** | **0.59** | **0.68** | **0.45** |
| **SD** | **0.15** | **0.04** | **0.05** | **0.06** | **0.07** | **0.15** | **0.10** |

Figure 5

Immunoblotting

1. The ratios of p-CREB/CREB in the cortical penumbra among the experimental groups at 7 d of reperfusion

|  | Sham | Vehicle | B-FA | P-FA | I-FA | R-FA | D-FA |
| --- | --- | --- | --- | --- | --- | --- | --- |
| 1 | 0.71 | 0.22 | 0.17 | 0.52 | 0.58 | 0.41 | 0.10 |
| 2 | 0.74 | 0.25 | 0.23 | 0.50 | 0.42 | 0.82 | 0.34 |
| 3 | 0.46 | 0.20 | 0.31 | 0.70 | 0.60 | 0.82 | 0.43 |
| 4 | 0.80 | 0.23 | 0.39 | 0.85 | 0.65 | 0.52 | 0.51 |
| **Mean** | **0.68** | **0.23** | **0.28** | **0.64** | **0.56** | **0.64** | **0.35** |
| **SD** | **0.15** | **0.02** | **0.10** | **0.17** | **0.10** | **0.21** | **0.18** |

2. The ratios of cytosolic Bcl-2/actin in the cortical penumbra among the experimental groups at 7 d of reperfusion

|  | Sham | Vehicle | B-FA | P-FA | I-FA | R-FA | D-FA |
| --- | --- | --- | --- | --- | --- | --- | --- |
| 1 | 0.45 | 0.25 | 0.19 | 0.47 | 0.48 | 0.43 | 0.49 |
| 2 | 0.38 | 0.13 | 0.11 | 0.56 | 0.74 | 0.77 | 0.29 |
| 3 | 0.41 | 0.15 | 0.24 | 0.52 | 0.58 | 0.58 | 0.42 |
| 4 | 0.48 | 0.20 | 0.41 | 0.38 | 0.88 | 0.55 | 0.24 |
| **5** | 0.56 | 0.15 | 0.29 | 0.49 | 0.70 | 0.51 | 0.36 |
| **Mean** | **0.46** | **0.18** | **0.25** | **0.48** | **0.68** | **0.57** | **0.36** |
| **SD** | **0.07** | **0.05** | **0.11** | **0.07** | **0.15** | **0.13** | **0.10** |

3. The ratios of cytosolic Bcl-2/Bax in the cortical penumbra among the experimental groups at 7 d of reperfusion

|  | Sham | Vehicle | B-FA | P-FA | I-FA | R-FA | D-FA |
| --- | --- | --- | --- | --- | --- | --- | --- |
| 1 | 2.48 | 0.95 | 0.71 | 1.18 | 2.10 | 1.42 | 1.00 |
| 2 | 1.95 | 0.41 | 0.35 | 2.16 | 3.74 | 3.45 | 0.83 |
| 3 | 2.40 | 0.52 | 0.89 | 2.64 | 2.17 | 2.32 | 1.82 |
| 4 | 1.66 | 0.50 | 0.97 | 1.70 | 2.06 | 2.00 | 0.60 |
| **5** | 1.41 | 0.50 | 1.19 | 1.73 | 2.01 | 2.22 | 1.11 |
| **Mean** | **1.98** | **0.58** | **0.82** | **1.88** | **2.42** | **2.28** | **1.07** |
| **SD** | **0.46** | **0.21** | **0.32** | **0.55** | **0.74** | **0.74** | **0.46** |

4. The ratios of cytosolic Bcl-xL/actin in the cortical penumbra among the experimental groups at 7 d of reperfusion

|  | Sham | Vehicle | B-FA | P-FA | I-FA | R-FA | D-FA |
| --- | --- | --- | --- | --- | --- | --- | --- |
| 1 | 0.46 | 0.32 | 0.34 | 0.52 | 0.66 | 0.55 | 0.37 |
| 2 | 0.67 | 0.25 | 0.33 | 0.78 | 0.76 | 1.01 | 0.72 |
| 3 | 0.63 | 0.43 | 0.59 | 0.71 | 1.03 | 0.67 | 0.63 |
| 4 | 0.72 | 0.35 | 0.42 | 0.59 | 0.96 | 0.81 | 0.78 |
| **Mean** | **0.62** | **0.34** | **0.42** | **0.65** | **0.85** | **0.76** | **0.63** |
| **SD** | **0.11** | **0.07** | **0.12** | **0.12** | **0.17** | **0.20** | **0.18** |

5. The ratios of cytosolic Bcl-xL/Bax in the cortical penumbra among the experimental groups at 7 d of reperfusion

|  | Sham | Vehicle | B-FA | P-FA | I-FA | R-FA | D-FA |
| --- | --- | --- | --- | --- | --- | --- | --- |
| 1 | 2.23 | 0.97 | 1.03 | 1.93 | 3.23 | 2.39 | 1.00 |
| 2 | 2.66 | 0.88 | 0.81 | 2.61 | 1.84 | 2.65 | 2.08 |
| 3 | 2.10 | 0.98 | 1.38 | 3.00 | 2.32 | 2.90 | 1.54 |
| 4 | 2.16 | 0.88 | 1.37 | 1.68 | 2.21 | 2.86 | 1.90 |
| **Mean** | **2.29** | **0.93** | **1.15** | **2.31** | **2.40** | **2.70** | **1.63** |
| **SD** | **0.25** | **0.06** | **0.28** | **0.61** | **0.59** | **0.23** | **0.48** |

Figure 6

Immunoblotting

1. The ratios of mitochondrial Bcl-2/Bax in the cortical penumbra among the experimental groups at 7 d of reperfusion

|  | Sham | Vehicle | B-FA | P-FA | I-FA | R-FA | D-FA |
| --- | --- | --- | --- | --- | --- | --- | --- |
| 1 | 1.36 | 0.21 | 0.26 | 0.71 | 1.09 | 0.87 | 0.48 |
| 2 | 0.70 | 0.12 | 0.06 | 0.72 | 0.90 | 0.86 | 0.32 |
| 3 | 0.80 | 0.22 | 0.30 | 0.73 | 0.74 | 0.87 | 0.31 |
| 4 | 1.20 | 0.21 | 0.31 | 0.70 | 0.50 | 0.67 | 0.15 |
| **5** | 0.69 | 0.20 | 0.81 | 1.07 | 1.02 | 1.02 | 0.71 |
| **Mean** | **0.95** | **0.19** | **0.35** | **0.79** | **0.85** | **0.86** | **0.39** |
| **SD** | **0.31** | **0.04** | **0.28** | **0.16** | **0.24** | **0.12** | **0.21** |

2. The ratios of mitochondrial Bcl-xL/Bax in the cortical penumbra among the experimental groups at 7 days of reperfusion

|  | Sham | Vehicle | B-FA | P-FA | I-FA | R-FA | D-FA |
| --- | --- | --- | --- | --- | --- | --- | --- |
| 1 | 1.98 | 1.77 | 1.57 | 3.25 | 3.96 | 3.63 | 1.27 |
| 2 | 2.48 | 1.41 | 1.58 | 2.06 | 2.13 | 1.80 | 1.02 |
| 3 | 1.85 | 1.70 | 1.34 | 2.42 | 3.93 | 2.63 | 2.25 |
| 4 | 1.96 | 1.03 | 1.55 | 3.01 | 1.20 | 2.69 | 1.06 |
| **Mean** | **2.07** | **1.48** | **1.51** | **2.69** | **2.81** | **2.69** | **1.40** |
| **SD** | **0.28** | **0.34** | **0.11** | **0.54** | **1.37** | **0.75** | **0.58** |

3. The expression of mitochondrial Bax/HSP60 in the cortical penumbra among the experimental groups at 7 days of reperfusion

|  | Sham | Vehicle | B-FA | P-FA | I-FA | R-FA | D-FA |
| --- | --- | --- | --- | --- | --- | --- | --- |
| 1 | 0.40 | 0.81 | 0.70 | 0.39 | 0.24 | 0.30 | 0.73 |
| 2 | 0.41 | 0.79 | 0.81 | 0.43 | 0.43 | 0.48 | 1.08 |
| 3 | 0.47 | 0.77 | 0.83 | 0.40 | 0.52 | 0.38 | 0.55 |
| 4 | 0.28 | 0.88 | 0.61 | 0.34 | 0.59 | 0.49 | 1.35 |
| **5** | 0.64 | 0.90 | 0.42 | 0.23 | 0.36 | 0.38 | 0.93 |
| **Mean** | **0.44** | **0.83** | **0.67** | **0.36** | **0.43** | **0.41** | **0.93** |
| **SD** | **0.13** | **0.06** | **0.17** | **0.08** | **0.14** | **0.08** | **0.31** |

4. The expression of mitochondrial AIF/HSP60 in the cortical penumbra among the experimental groups at 7 days of reperfusion

|  | Sham | Vehicle | B-FA | P-FA | I-FA | R-FA | D-FA |
| --- | --- | --- | --- | --- | --- | --- | --- |
| 1 | 0.19 | 0.88 | 0.80 | 0.75 | 0.66 | 0.74 | 0.31 |
| 2 | 0.84 | 1.16 | 0.36 | 0.60 | 0.78 | 0.50 | 0.34 |
| 3 | 0.92 | 1.34 | 1.01 | 0.75 | 1.41 | 0.73 | 1.34 |
| 4 | 0.14 | 0.52 | 0.56 | 1.19 | 0.17 | 1.58 | 0.88 |
| **5** | 0.32 | 0.78 | 0.29 | 0.72 | 0.69 | 0.56 | 0.22 |
| **Mean** | **0.48** | **0.94** | **0.60** | **0.80** | **0.74** | **0.82** | **0.62** |
| **SD** | **0.37** | **0.32** | **0.30** | **0.23** | **0.44** | **0.44** | **0.48** |

5. The ratios of cleaved caspase-3/actin in the cortical penumbra among the experimental groups at 7 d of reperfusion

|  | Sham | Vehicle | B-FA | P-FA | I-FA | R-FA | D-FA |
| --- | --- | --- | --- | --- | --- | --- | --- |
| 1 | 0.14 | 0.32 | 0.24 | 0.10 | 0.14 | 0.19 | 0.64 |
| 2 | 0.14 | 0.39 | 0.31 | 0.14 | 0.11 | 0.11 | 0.38 |
| 3 | 0.10 | 0.35 | 0.30 | 0.13 | 0.08 | 0.14 | 0.34 |
| 4 | 0.12 | 0.44 | 0.54 | 0.16 | 0.14 | 0.14 | 0.52 |
| **Mean** | **0.13** | **0.38** | **0.35** | **0.13** | **0.12** | **0.15** | **0.47** |
| **SD** | **0.02** | **0.05** | **0.13** | **0.03** | **0.03** | **0.03** | **0.14** |

6. The ratios of AIF/actin in the cortical penumbra among the experimental groups at 7 d of reperfusion

|  | Sham | Vehicle | B-FA | P-FA | I-FA | R-FA | D-FA |
| --- | --- | --- | --- | --- | --- | --- | --- |
| 1 | 0.16 | 0.24 | 0.73 | 0.24 | 0.34 | 0.37 | 0.31 |
| 2 | 0.19 | 0.20 | 0.26 | 0.26 | 0.35 | 0.35 | 0.36 |
| 3 | 0.20 | 0.31 | 0.21 | 0.16 | 0.26 | 0.47 | 0.34 |
| 4 | 0.35 | 0.18 | 0.16 | 0.29 | 0.69 | 0.31 | 0.44 |
| **5** | 0.39 | 0.34 | 0.79 | 0.47 | 0.66 | 0.50 | 0.40 |
| **Mean** | **0.26** | **0.25** | **0.43** | **0.28** | **0.46** | **0.40** | **0.37** |
| **SD** | **0.10** | **0.07** | **0.30** | **0.11** | **0.20** | **0.08** | **0.05** |

Figure 7

IHC

1. The number of cytochrome c immunopositive cells in the cortical penumbra among the experimental groups at 7 d after reperfusion

|  | Sham | vehicle | B-FA | P-FA | I-FA | R-FA | D-FA |
| --- | --- | --- | --- | --- | --- | --- | --- |
| 1 | 0 | 103 | 116 | 54 | 32 | 24 | 104 |
| 2 | 0 | 130 | 136 | 47 | 50 | 20 | 99 |
| 3 | 0 | 121 | 179 | 56 | 40 | 29 | 162 |
| **Mean** | **0** | **118** | **144** | **52** | **41** | **24** | **122** |
| **SD** | **0** | **14** | **32** | **5** | **9** | **5** | **35** |

2. The number of cleaved caspase-3 immunopositive cells in the cortical penumbra among the experimental groups at 7 d after reperfusion

|  | Sham | vehicle | B-FA | P-FA | I-FA | R-FA | D-FA |
| --- | --- | --- | --- | --- | --- | --- | --- |
| 1 | 0 | 125 | 168 | 72 | 46 | 36 | 185 |
| 2 | 0 | 149 | 183 | 56 | 39 | 37 | 161 |
| 3 | 0 | 187 | 194 | 89 | 44 | 30 | 145 |
| **Mean** | **0** | **154** | **182** | **72** | **43** | **34** | **164** |
| **SD** | **0** | **31** | **13** | **17** | **4** | **4** | **20** |

Figure 9

The percentage of infarct area was calculated at 7 d of reperfusion

|  | D+Sham | D+vehicle | D+I-FA | SB+I-FA |  |  |  |
| --- | --- | --- | --- | --- | --- | --- | --- |
| 1 | 0 | 17.5 | 9.9 | 20.7 |  |  |  |
| 2 | 0 | 20.0 | 13.4 | 26.0 |  |  |  |
| 3 | 0 | 25.1 | 11.3 | 18.7 |  |  |  |
| **Mean** | **0** | **20.9** | **11.5** | **21.8** |  |  |  |
| **SD** | **0** | **3.9** | **1.8** | **3.8** |  |  |  |

Figure 10

Immunoblotting

1. The ratios of p-p38 MAPK/p38 MAPK in the cortical penumbra among the experimental groups at 7 d of reperfusion

|  | D+Sham | D+Vehicle | D+I-FA | SB+I-FA |  |  |  |
| --- | --- | --- | --- | --- | --- | --- | --- |
| 1 | 0.54 | 0.20 | 0.66 | 0.18 |  |  |  |
| 2 | 0.67 | 0.06 | 1.04 | 0.19 |  |  |  |
| 3 | 0.73 | 0.14 | 0.58 | 0.29 |  |  |  |
| 4 | 0.67 | 0.10 | 0.73 | 0.31 |  |  |  |
| **5** | 0.55 | 0.10 | 0.80 | 0.44 |  |  |  |
| **Mean** | **0.63** | **0.12** | **0.76** | **0.28** |  |  |  |
| **SD** | **0.08** | **0.05** | **0.18** | **0.11** |  |  |  |

2. The ratios of p-p90RSK/actin in the cortical penumbra among the experimental groups at 7 d of reperfusion

|  | D+Sham | D+Vehicle | D+I-FA | SB+I-FA |  |  |  |
| --- | --- | --- | --- | --- | --- | --- | --- |
| 1 | 0.42 | 0.13 | 0.18 | 0.06 |  |  |  |
| 2 | 0.29 | 0.11 | 0.58 | 0.07 |  |  |  |
| 3 | 0.34 | 0.14 | 0.30 | 0.11 |  |  |  |
| 4 | 0.38 | 0.15 | 0.25 | 0.08 |  |  |  |
| **5** | 0.24 | 0.13 | 0.50 | 0.17 |  |  |  |
| **Mean** | **0.33** | **0.13** | **0.36** | **0.10** |  |  |  |
| **SD** | **0.07** | **0.01** | **0.17** | **0.04** |  |  |  |

3. The ratios of p-Bad/actin in the cortical penumbra among the experimental groups at 7 d of reperfusion

|  | D+Sham | D+Vehicle | D+I-FA | SB+I-FA |  |  |  |
| --- | --- | --- | --- | --- | --- | --- | --- |
| 1 | 0.41 | 0.19 | 0.34 | 0.14 |  |  |  |
| 2 | 0.28 | 0.11 | 0.32 | 0.10 |  |  |  |
| 3 | 0.32 | 0.18 | 0.37 | 0.10 |  |  |  |
| 4 | 0.33 | 0.17 | 0.35 | 0.14 |  |  |  |
| **5** | 0.32 | 0.16 | 0.32 | 0.13 |  |  |  |
| **Mean** | **0.33** | **0.16** | **0.34** | **0.12** |  |  |  |
| **SD** | **0.05** | **0.03** | **0.02** | **0.02** |  |  |  |

4. The ratios of p-CREB/CREB in the cortical penumbra among the experimental groups at 7 d of reperfusion

|  | D+Sham | D+Vehicle | D+I-FA | SB+I-FA |  |  |  |
| --- | --- | --- | --- | --- | --- | --- | --- |
| 1 | 0.55 | 0.23 | 0.53 | 0.10 |  |  |  |
| 2 | 0.41 | 0.14 | 0.87 | 0.31 |  |  |  |
| 3 | 0.86 | 0.15 | 0.42 | 0.39 |  |  |  |
| 4 | 0.43 | 0.17 | 0.73 | 0.40 |  |  |  |
| **5** | 0.53 | 0.11 | 0.77 | 0.26 |  |  |  |
| **Mean** | **0.56** | **0.16** | **0.66** | **0.29** |  |  |  |
| **SD** | **0.18** | **0.04** | **0.18** | **0.12** |  |  |  |

5. The ratios of cytosolic Bcl-2/Bax in the cortical penumbra among the experimental groups at 7 d of reperfusion

|  | D+Sham | D+Vehicle | D+I-FA | SB+I-FA |  |  |  |
| --- | --- | --- | --- | --- | --- | --- | --- |
| 1 | 1.46 | 0.67 | 1.58 | 0.48 |  |  |  |
| 2 | 1.30 | 0.17 | 1.95 | 0.39 |  |  |  |
| 3 | 1.09 | 0.31 | 1.55 | 0.45 |  |  |  |
| 4 | 1.89 | 0.41 | 1.00 | 0.56 |  |  |  |
| **5** | 1.18 | 0.41 | 1.36 | 0.49 |  |  |  |
| **Mean** | **1.38** | **0.39** | **1.49** | **0.47** |  |  |  |
| **SD** | **0.32** | **0.18** | **0.35** | **0.06** |  |  |  |

Figure 11

Immunoblotting

1. The ratios of mitochondrial Bcl-2/Bax in the cortical penumbra among the experimental groups at 7 d of reperfusion

|  | D+Sham | D+Vehicle | D+I-FA | SB+I-FA |  |  |  |
| --- | --- | --- | --- | --- | --- | --- | --- |
| 1 | 1.87 | 0.25 | 0.60 | 0.07 |  |  |  |
| 2 | 1.94 | 0.14 | 0.66 | 0.20 |  |  |  |
| 3 | 1.29 | 0.15 | 0.98 | 0.25 |  |  |  |
| 4 | 1.44 | 0.28 | 1.03 | 0.20 |  |  |  |
| **5** | 0.74 | 0.30 | 1.28 | 0.55 |  |  |  |
| **Mean** | **1.46** | **0.22** | **0.91** | **0.25** |  |  |  |
| **SD** | **0.49** | **0.07** | **0.28** | **0.18** |  |  |  |

2. The ratios of mitochondrial Bax/HSP60 in the cortical penumbra among the experimental groups at 7 d of reperfusion

|  | D+Sham | D+Vehicle | D+I-FA | SB+I-FA |  |  |  |
| --- | --- | --- | --- | --- | --- | --- | --- |
| 1 | 0.32 | 0.64 | 0.30 | 0.42 |  |  |  |
| 2 | 0.12 | 0.69 | 0.33 | 0.57 |  |  |  |
| 3 | 0.25 | 0.67 | 0.50 | 0.48 |  |  |  |
| 4 | 0.35 | 0.65 | 0.33 | 0.28 |  |  |  |
| **5** | 0.41 | 0.61 | 0.40 | 0.65 |  |  |  |
| **Mean** | **0.29** | **0.65** | **0.37** | **0.48** |  |  |  |
| **SD** | **0.11** | **0.03** | **0.08** | **0.14** |  |  |  |

3. The ratios of cleaved caspase-3/actin in the cortical penumbra among the experimental groups at 7 d of reperfusion

|  | D+Sham | D+Vehicle | D+I-FA | SB+I-FA |  |  |  |
| --- | --- | --- | --- | --- | --- | --- | --- |
| 1 | 0.16 | 0.45 | 0.18 | 0.26 |  |  |  |
| 2 | 0.15 | 0.48 | 0.15 | 0.23 |  |  |  |
| 3 | 0.11 | 0.43 | 0.15 | 0.45 |  |  |  |
| 4 | 0.15 | 0.36 | 0.13 | 0.25 |  |  |  |
| **5** | 0.27 | 0.50 | 0.21 | 0.49 |  |  |  |
| **Mean** | **0.17** | **0.44** | **0.16** | **0.34** |  |  |  |
| **SD** | **0.06** | **0.05** | **0.03** | **0.12** |  |  |  |
